# Assessing the Accuracy of Generative Conversational Artificial Intelligence in Debunking Sleep Health Myths: Mixed Methods Comparative Study With Expert Analysis

**DOI:** 10.2196/55762

**Published:** 2024-04-16

**Authors:** Nicola Luigi Bragazzi, Sergio Garbarino

**Affiliations:** 1 Human Nutrition Unit Department of Food and Drugs University of Parma Parma Italy; 2 Department of Neuroscience, Rehabilitation, Ophthalmology, Genetics and Maternal/Child Sciences University of Genoa Genoa Italy; 3 Laboratory for Industrial and Applied Mathematics Department of Mathematics and Statistics York University Toronto, ON Canada; 4 Post-Graduate School of Occupational Health Università Cattolica del Sacro Cuore Rome Italy

**Keywords:** sleep, sleep health, sleep-related disbeliefs, generative conversational artificial intelligence, chatbot, ChatGPT, misinformation, artificial intelligence, comparative study, expert analysis, adequate sleep, well-being, sleep trackers, sleep health education, sleep-related, chronic disease, healthcare cost, sleep timing, sleep duration, presleep behaviors, sleep experts, healthy behavior, public health, conversational agents

## Abstract

**Background:**

Adequate sleep is essential for maintaining individual and public health, positively affecting cognition and well-being, and reducing chronic disease risks. It plays a significant role in driving the economy, public safety, and managing health care costs. Digital tools, including websites, sleep trackers, and apps, are key in promoting sleep health education. Conversational artificial intelligence (AI) such as ChatGPT (OpenAI, Microsoft Corp) offers accessible, personalized advice on sleep health but raises concerns about potential misinformation. This underscores the importance of ensuring that AI-driven sleep health information is accurate, given its significant impact on individual and public health, and the spread of sleep-related myths.

**Objective:**

This study aims to examine ChatGPT’s capability to debunk sleep-related disbeliefs.

**Methods:**

A mixed methods design was leveraged. ChatGPT categorized 20 sleep-related myths identified by 10 sleep experts and rated them in terms of falseness and public health significance, on a 5-point Likert scale. Sensitivity, positive predictive value, and interrater agreement were also calculated. A qualitative comparative analysis was also conducted.

**Results:**

ChatGPT labeled a significant portion (n=17, 85%) of the statements as “false” (n=9, 45%) or “generally false” (n=8, 40%), with varying accuracy across different domains. For instance, it correctly identified most myths about “sleep timing,” “sleep duration,” and “behaviors during sleep,” while it had varying degrees of success with other categories such as “pre-sleep behaviors” and “brain function and sleep.” ChatGPT’s assessment of the degree of falseness and public health significance, on the 5-point Likert scale, revealed an average score of 3.45 (SD 0.87) and 3.15 (SD 0.99), respectively, indicating a good level of accuracy in identifying the falseness of statements and a good understanding of their impact on public health. The AI-based tool showed a sensitivity of 85% and a positive predictive value of 100%. Overall, this indicates that when ChatGPT labels a statement as false, it is highly reliable, but it may miss identifying some false statements. When comparing with expert ratings, high intraclass correlation coefficients (ICCs) between ChatGPT’s appraisals and expert opinions could be found, suggesting that the AI’s ratings were generally aligned with expert views on falseness (ICC=.83, *P*<.001) and public health significance (ICC=.79, *P*=.001) of sleep-related myths. Qualitatively, both ChatGPT and sleep experts refuted sleep-related misconceptions. However, ChatGPT adopted a more accessible style and provided a more generalized view, focusing on broad concepts, while experts sometimes used technical jargon, providing evidence-based explanations.

**Conclusions:**

ChatGPT-4 can accurately address sleep-related queries and debunk sleep-related myths, with a performance comparable to sleep experts, even if, given its limitations, the AI cannot completely replace expert opinions, especially in nuanced and complex fields such as sleep health, but can be a valuable complement in the dissemination of updated information and promotion of healthy behaviors.

## Introduction

An adequate amount of good, restorative sleep is of paramount importance for both individual and public health [1,2]: from an individual standpoint, it helps maintain optimal physical and mental health, facilitating cognitive function, ensuring well-being, and mitigating the risks associated with chronic diseases [3]. In the context of public health, sleep’s impact is profound and multifaceted as well, being a pivotal element in driving the economy, ensuring public safety, and managing health care expenditures. The strategic addressing of sleep-related issues not only alleviates the global burden of disease but also ameliorates the economic strain associated with it [4,5].

The promotion of healthy sleep patterns and the intervention in sleep-related disorders emerge as vital strategies, paving the way for the enhancement of overall societal well-being, boosting productivity, and fostering social cohesion [6]. Such initiatives can yield substantial benefits, at both the individual and community levels, thereby underscoring the role of innovative tools, including digital ones—spanning from dynamic websites to sleep trackers and mobile apps—in promoting and providing education on sleep health [7].

The internet offers a vast, versatile, easily accessible, and cost-effective platform for disseminating up-to-date information about sleep, reaching diverse populations, raising public awareness about the importance of sleep, and providing personalized guidance on sleep health and related topics. People can access the latest findings and recommendations to make informed decisions about their sleep habits, with telemedicine and web-based consultations with sleep experts becoming increasingly popular. The digital realm can enable individuals to monitor their sleep patterns, engaging them in continuous learning about sleep health, and facilitating self-awareness and behavioral changes to improve sleep quality [8].

In the era of generative conversational artificial intelligence (AI) [9,10], characterized by disruptive technological transformation, the importance of sleep health promotion and education becomes even more relevant [11]: conversational AI-based platforms and agents, such as chatbots, can provide instant responses to sleep-related queries, making information readily available at any time. This real-time accessibility can help individuals seeking answers about sleep health, who can receive personalized advice and recommendations based on an individual’s specific sleep patterns and concerns. However, besides being accessible and tailored, this information should also be accurate [12].

There are only a few studies that have assessed sleep-related knowledge of conversational AI-based chatbots, such as ChatGPT-4, which was found very recently to successfully pass the sleep medicine certification board examinations [13] and be conversant in sleep disorders, such as obstructive sleep apnea syndrome [14-16].

On the other hand, conversational AI may contribute to disseminating “factual errors, nonsense, fabricated sources, and dangerous advice” and, thus, spreading biomedical misinformation, including sleep-related misinformation [17]. Therefore, our study was conducted to verify the accuracy of a popular prototype of conversational AI, ChatGPT, in addressing queries concerning sleep health and, in particular, sleep-related myths. These can be defined as widely held “false beliefs about sleep” that “lack an evidence base” and “can persist despite contradicting scientific evidence, potentially impairing” and even degrading population health, by promoting the adoption of unhealthy behaviors and lifestyles, the identification of which “can inform efforts to promote population sleep health” [18].

## Methods

### Procedure

A list of 20 sleep-related myths, as defined above, was taken from a previously published study [18]. This list was compiled using internet searches of popular press and scientific literature and leveraging a Delphi process that involved 10 sleep experts from the fields of sleep medicine and research. Experts were recruited by convenience sampling, after being identified through literature searches (using PubMed). To be considered an expert, they were required to have published 20 papers that were cited by 20 or more different peer-reviewed sources, and at least one of these publications had to be tagged with the “Medical Subject Headings” “sleep” along with either “circadian rhythms,” “neuroscience,” or “psychiatry.” A total of 20 individuals who fulfilled these requirements were reached out to and out of these 20 experts 10 took part in this study. The Delphi process consisted of selecting and refining myths and was conducted in 3 stages: initially, focus groups were held (phase 1); this was followed by a period of email-based feedback for editing, adding, or removing myths (phase 2); finally, closed-ended surveys were used (phase 3), during which experts assessed the myths. The 20 myths were, then, categorized along six domains: namely, (1) “sleep duration” (n=6), (2) “sleep timing” (n=1), (3) “behaviors during sleep” (n=4), (4) “daytime behaviors that relate to sleep” (n=2), (5) “pre-sleep behaviors” (n=5), and (6) “brain function and sleep” (n=2). Besides providing feedback, experts had to rate myths on 2 dimensions: falseness and public health significance using a 5-point Likert scale from 1 (“not at all false” or “not at all significant”) to 5 (“extremely false” or “extremely significant”) [18].

It should be noted that, while some of these myths are patently false (such as the statement “during sleep, the brain is not active,” which belongs to the “brain function and sleep” domain), other statements such as “lying in bed with your eyes closed is almost as good as sleeping” (belonging to the “behaviors during sleep” domain) contain some elements of truth and other sleep experts may disagree in labeling them as complete misinformation or myths. Indeed, whether “waking rest” and other “resting states” may confer benefits almost as good as deep rest is debated [19,20]: these concepts challenge the conventional dichotomy of sleep and wakefulness conceived as binary and distinct states and suggest that periods of quiet reflection during wakefulness, characterized by a lack of effortful, focused thought and the absence of distracting stimuli, can also contribute significantly to mental rejuvenation, memory consolidation, hormonal regulation, cellular repair, and emotion regulation [21-23]. Altogether, these resting states, defined also as “offline states,” including eyes-closed rest, daydreaming, mind wandering, or inattentive states, represent approximately half of our waking hours [24,25]. However, the existing scholarly literature reports scarce, contrasting, or even negative findings [26], that warrant further research and suggest that these concepts are not yet well established and are continuously evolving [19,20].

Sleep is, indeed, a complex, nonlinear process, and sometimes, our perception of how well we sleep or even whether we are asleep or awake can be incorrect. For instance, “sleep state misperception,” also known as “paradoxical insomnia,” is a condition where individuals believe they are awake for most of the night, despite actually sleeping for a normal duration. This disorder is characterized by a significant discrepancy between perceived and actual sleep time, often associated with personality traits like neuroticism and altered brain activity during sleep, though its causes and prevalence remain under investigation [27-30].

### Ethical Considerations

Full ethical clearance was waived for this study, as this study is a purely observational study with responses generated by AI (ChatGPT) and secondary analysis of research data, consisting of anonymous or deidentified study data [18].

### Statistical Analysis

ChatGPT (version 4) was asked both to determine if these 20 sleep-related disbeliefs were true or false and to appraise them using the 5-point Likert scale. To determine if there is a statistical difference between the 6 abovementioned domains in terms of the distribution of true, false, and other categories of responses, a chi-square test of independence was used. This test helped us determine if the differences in proportions across the different domains were statistically significant. Means were also reported for the overall score (along with their SDs) and broken down according to each domain.

In terms of accuracy, the sensitivity and the positive predictive value of ChatGPT in categorizing the sleep-related statements as false were computed.

Finally, ChatGPT’s ratings of falseness and public health significance of sleep-related myths were compared with those provided by sleep experts. The degree of agreement was measured, in terms of consistency, using the interrater reliability analysis, computing the intraclass correlation coefficients (ICCs) [31].

All statistical analyses were carried out using SPSS (version 28 for Windows; IBM Corp). *P* values less than .05 were considered statistically significant.

### Qualitative Analytical Approach

A qualitative comparative analysis was also conducted. Initially, responses from ChatGPT and summary responses from the sleep experts [18] were subjected to a line-by-line comparison to identify similarities and differences in content, style, and complexity of information provided. Then, responses were scrutinized to identify themes, concepts, or categories that were entered in a matrix to have a clear snapshot of where ChatGPT and the experts aligned or diverged in their discussions and to make emerging patterns of alignment and divergence between them. This phase was crucial for understanding how ChatGPT’s training data correlated or not with the current consensus among experts and enabled the identification of gaps in ChatGPT’s knowledge base.

## Results

### ChatGPT’s Falseness Quantitative Analysis of Sleep-Related Myths

Overall, ChatGPT labeled 45% (n=9) of the statements as “false,” while a further 40% (n=8) of the items were deemed as “generally false.” Of note, concerning the remaining statements, 5% (n=1) and 10% (n=2) of them were considered “true” and “not (entirely or necessarily) true or false,” respectively. In terms of domain, half of the items related to “sleep duration” were considered “false” (n=3), with the remaining half percent being deemed “generally false” (n=3). The statement concerning “sleep timing” was labeled as “generally false” (n=1). Further, 75% (n=3) of the items related to “behaviors during sleep” were correctly identified as “false,” while the remaining 25% (n=1) were classified as “generally false.” All the statements concerning “daytime behaviors related to sleep” were considered “generally false” (n=2). When assessing the accuracy of items concerning “pre-sleep behaviors,” half of them were properly labeled as “false” (n=2), whereas 16.67% (n=1) of the statements were considered “generally false,” with a further 16.67% (n=1) being “not entirely true or false” and the remaining 16.67% (n=1) being even considered “true.” Finally, concerning “brain function and sleep,” half of the statements were correctly appraised as “false” (n=1), with the remaining half being labeled as “not necessarily true or false” (n=1). Further details are reported in Table 1 and Multimedia Appendix 1.

**Table 1:** ChatGPT’s appraisals of the falseness of sleep-related myths, scored both qualitatively (true or false) and quantitatively (on a 5-point Likert scale, from 1 or “not at all false” to 5 or “extremely false”), encompassing a range of 6 different topics (sleep duration, sleep timing, behaviors during sleep, daytime behaviors that relate to sleep, presleep behaviors, and brain function and sleep).

Sleep-related myths			ChatGPT, mean		
			True or false		On the 5-point Likert scale
**Sleep duration**					
	“Being able to fall asleep ‘anytime, anywhere’ is a sign of a healthy sleep system”	Generally false		4.5	
	“Many adults need only 5 or less hours of sleep for general health”	False		4.5	
	“Your brain and body can learn to function just as well with less sleep”	False		4	
	“Adults sleep more as they get older”	Generally false		4	
	“If you can get it, more sleep is always better”	False		3	
	“One night of sleep deprivation will have lasting negative health consequences”	Generally false		2	
**Sleep timing**					
	“In terms of your health, it does not matter what time of day you sleep”	Generally false		4	
**Behaviors during sleep**					
	“Lying in bed with your eyes closed is almost as good as sleeping”	False		4	
	“If you have difficulty falling asleep, it is best to stay in bed and try to fall back to sleep”	Generally false		3	
	“Although annoying for bed partners, loud snoring is mostly harmless”	False		3	
	“A sound sleeper rarely moves at night”	False		3	
**Daytime behaviors that relate to sleep**					
	“Hitting the snooze when you wake up is better than getting up when the alarm first goes off”	Generally false		4	
	“If you are having difficulties sleeping, taking a nap in the afternoon is a good way to get adequate sleep”	Generally false		2	
**Presleep behaviors**					
	“Alcohol before bed will improve your sleep”	False		4	
	“For sleeping, it is better to have a warmer bedroom than a cooler bedroom”	False		4	
	“Boredom can make you sleepy even if you got adequate sleep before”	True		2	
	“Watching television in bed is a good way to relax before sleep”	Generally false		3	
	“Exercising within 4 hours of bedtime will disturb your sleep”	Not entirely true or false		3	
**Brain function and sleep**					
	“During sleep, the brain is not active”	False		5	
	“Remembering your dreams is a sign of a good night’s sleep”	Not necessarily true or false		3	

The various response categories did not vary depending on the domain of sleep-related myths (*χ*^2^_15_=14.60, *P*=.48).

On the 5-point Likert scale, the degree of falseness was computed at 3.45 (SD 0.87), according to ChatGPT’s estimates. The highest scores were recorded for “brain function and sleep” (4.00, SD 1.41), “sleep timing” (4.00, single item), and “sleep duration” (3.67, SD 0.98), while the “behavioral domains” scored the lowest. More in detail, “behaviors during sleep” yielded a value of 3.25 (SD 0.50), followed by “pre-sleep behaviors” (3.20, SD 0.84) and “daytime behaviors that relate to sleep” (3.00, SD 1.41). Further details are presented in Table 1 and Multimedia Appendix 2.

Based on these data, ChatGPT demonstrated an overall sensitivity of 85% and a positive predictive value of 100% in categorizing the statements as “false.”

### Quantitative Comparison of ChatGPT’s and Expert Ratings on the Falseness of Sleep-Related Myths

**Figure 1:**
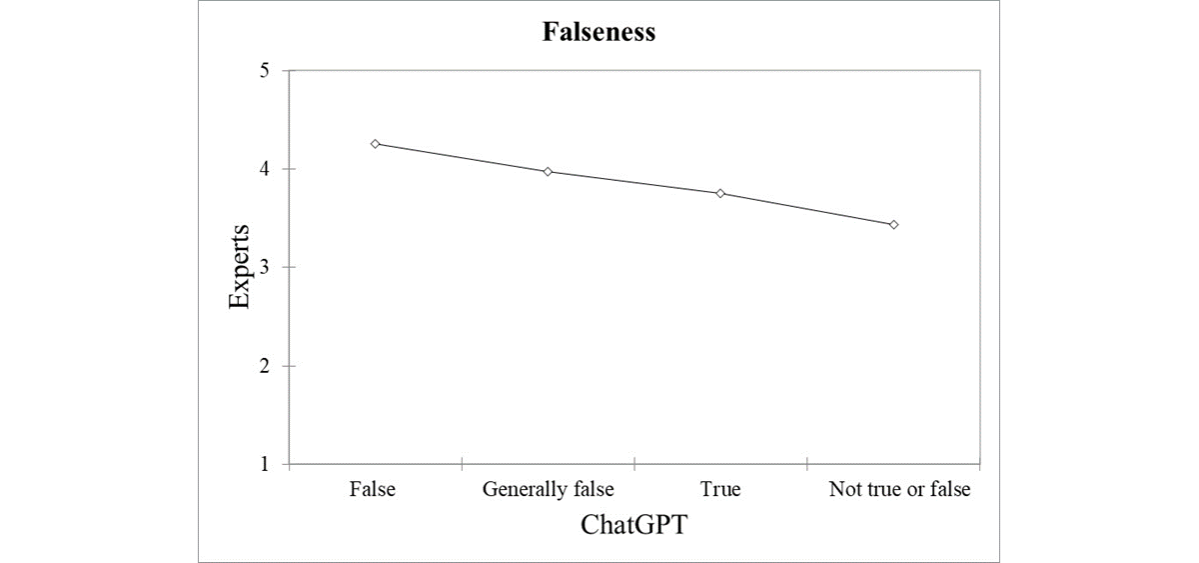
Comparison of ChatGPT’s and expert qualitative ratings on the falseness of sleep-related myths, showing a general good agreement and alignment between experts and artificial intelligence.

**Figure 2:**
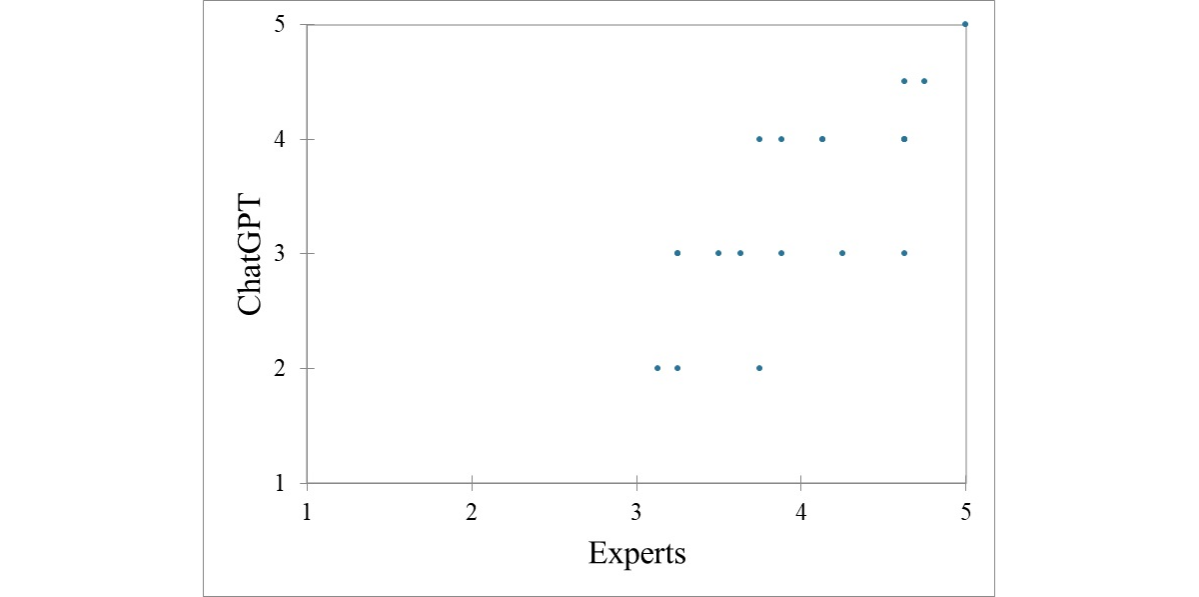
Scatterplot of the comparison of ChatGPT’s and expert quantitative ratings (on a 5-point Likert scale, from 1 or “not at all false” to 5 or “extremely false”) on the falseness of sleep-related myths, showing a general good agreement and alignment between experts and artificial intelligence.

When comparing with sleep experts, a good interrater agreement could be found between ChatGPT’s categorization of statements and expert rating on their falseness. Statements categorized by ChatGPT as “false” and “generally false” were those that received the highest scores by the experts (4.25 and 3.97, respectively), whereas those judged by the AI as “true” and “not true or false” received the lowest scores by the experts (3.75 and 3.44, respectively), as shown in Figure 1. From a more quantitative standpoint, the association yielded an ICC value of 0.83 (*P*<.001), when ChatGPT was asked to rate the degree of falseness of the statement on the 5-point Likert scale (Figure 2).

### ChatGPT’s Public Health Significance Quantitative Analysis of Sleep-Related Myths

The overall score was 3.15 (SD 0.99). “Sleep timing” was the domain scoring the highest (4.00, single item), followed by “sleep duration” (3.33, SD 1.37) and “behaviors during sleep” (3.25, SD 0.96). “Daytime behaviors that relate to sleep” scored the lowest (2.50, SD 0.71), while both “brain function and sleep” and “pre-sleep behaviors” yielded a mean score of 3.00 (SD 1.41, and SD 0.71, respectively). Further details are presented in Table 2.

**Table 2:** ChatGPT’s appraisals of the public health significance of sleep-related myths, scored quantitatively (on a 5-point Likert scale, from 1 or “not at all significant” to 5 or “extremely significant”), encompassing a range of 6 different topics (sleep duration, sleep timing, behaviors during sleep, daytime behaviors that relate to sleep, presleep behaviors, and brain function and sleep).

Sleep-related myths		ChatGPT (on the 5-point Likert scale), mean
**Sleep duration**		
	“Being able to fall asleep ‘anytime, anywhere’ is a sign of a healthy sleep system”	2
	“Many adults need only 5 or less hours of sleep for general health”	5
	“Your brain and body can learn to function just as well with less sleep”	5
	“Adults sleep more as they get older”	3
	“If you can get it, more sleep is always better”	3
	“One night of sleep deprivation will have lasting negative health consequences”	2
**Sleep timing**		
	“In terms of your health, it does not matter what time of day you sleep”	4
**Behaviors during sleep**		
	“Lying in bed with your eyes closed is almost as good as sleeping”	4
	“If you have difficulty falling asleep, it is best to stay in bed and try to fall back to sleep”	3
	“Although annoying for bed partners, loud snoring is mostly harmless”	4
	“A sound sleeper rarely moves at night”	2
**Daytime behaviors that relate to sleep**		
	“Hitting the snooze when you wake up is better than getting up when the alarm first goes off”	2
	“If you are having difficulties sleeping, taking a nap in the afternoon is a good way to get adequate sleep”	3
**Presleep behaviors**		
	“Alcohol before bed will improve your sleep”	4
	“For sleeping, it is better to have a warmer bedroom than a cooler bedroom”	3
	“Boredom can make you sleepy even if you got adequate sleep before”	2
	“Watching television in bed is a good way to relax before sleep”	3
	“Exercising within 4 hours of bedtime will disturb your sleep”	3
**Brain function and sleep**		
	“During sleep, the brain is not active”	4
	“Remembering your dreams is a sign of a good night’s sleep”	2

### Quantitative Comparison of ChatGPT’s and Expert Ratings on the Public Health Significance of Sleep-Related Myths

**Figure 3:**
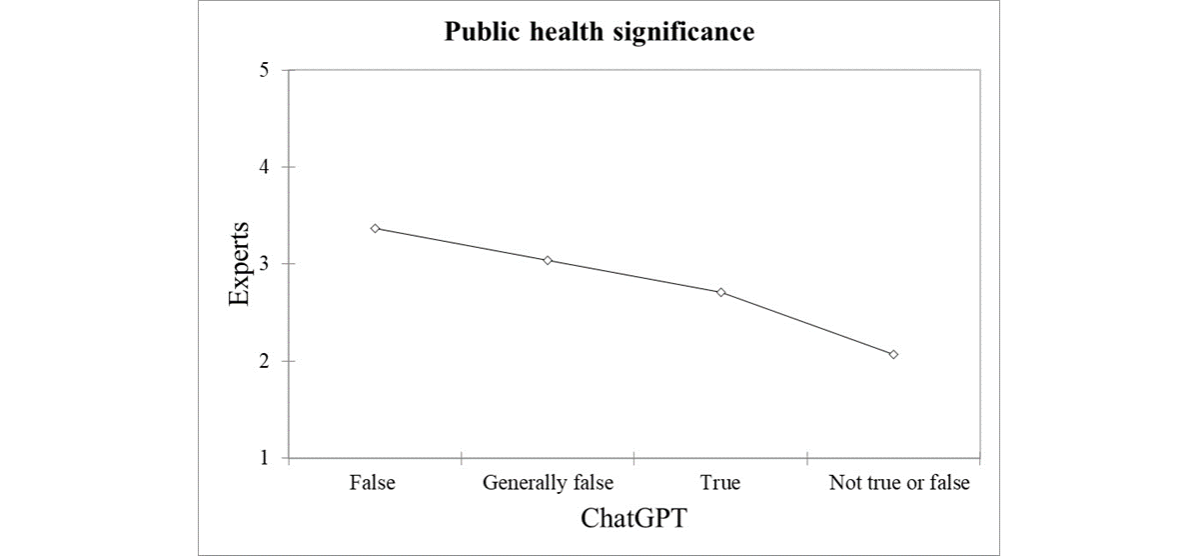
Comparison of ChatGPT’s and expert ratings on the public health significance of sleep-related myths, showing a general satisfactory agreement and alignment between experts and artificial intelligence.

Similar trends to those observed for the expert appraisals of the falseness of sleep-related myths could be reported for the expert rating on their public health significance. Items labeled by ChatGPT as “false” and “generally false” corresponded to a score of 3.37 and 3.04, respectively, while statements appraised by the AI as “true” and “not true or false” scored the lowest (2.71 and 2.07, respectively), as pictorially represented in Figure 3.

**Figure 4:**
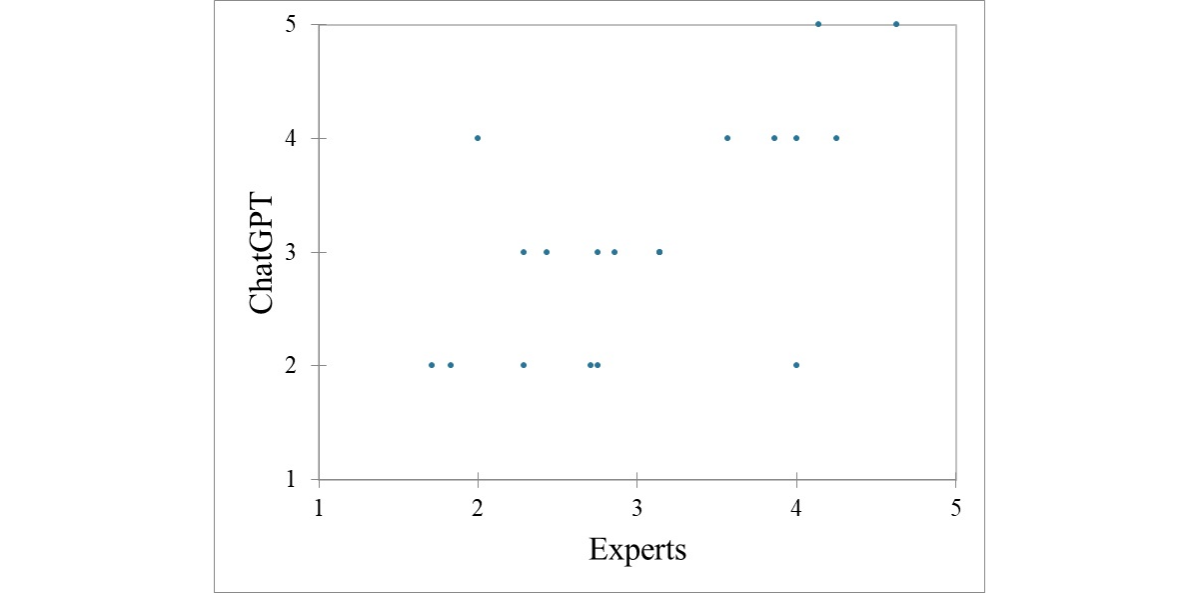
Scatterplot of the comparison of ChatGPT’s and expert quantitative ratings (on a 5-point Likert scale, from 1 or “not at all significant” to 5 or “extremely significant”) on the public health significance of sleep-related myths, showing a general satisfactory agreement and alignment between experts and artificial intelligence.

When comparing the ratings on public health significance provided by ChatGPT with those by the experts, an ICC of 0.79 could be computed (*P*=.001), as shown in Figure 4.

### Qualitative Comparison and Thematic Analysis of ChatGPT’s and Sleep Experts’ Responses

The qualitative comparison between ChatGPT’s and sleep experts’ appraisals of sleep-related myths revealed both similarities and differences across the various misconceptions.

In general, both ChatGPT and sleep experts clarified and, in some instances, strongly refuted these misconceptions, highlighting the importance of adequate sleep for overall well-being, while acknowledging individual differences in sleep needs and patterns, and emphasizing that sleep requirements can vary from person to person. However, differences in responses could be noted: ChatGPT adopted a more conversational and accessible style and provided a more generalized view, focusing on broad concepts, while experts sometimes used technical jargon, providing evidence-based explanations to debunk false beliefs about sleep. Sleep experts tended to provide a more detailed assessment, often including specific medical and physiological contexts, referencing studies, and focusing both on individual (clinical) and public health implications, while a population health perspective was generally missing in ChatGPT’s responses.

Specifically concerning “sleep duration myths,” both ChatGPT and sleep experts clarified the misconceptions, but experts provided a more medically oriented focus, delving into the biological and physiological underpinnings more deeply, while ChatGPT emphasized sleep-related flexibility and individual variability. Further, sleep experts provided a more nuanced appraisal of risks and the evidence (or lack thereof) supporting them, focusing not only on temporary or short-term effects but offering detailed perspectives on long-term consequences and recovery too. Similarly, concerning “sleep-related timing myths,” both ChatGPT and sleep experts emphasized the role of circadian rhythms and the importance of sleep timing, but experts gave a more technical assessment focusing on specific physiological processes. Regarding “behaviors during sleep” myths and “daytime sleep-related behaviors” myths, sleep experts delved into physiological, cultural, and habitual aspects and potential adverse outcomes. Concerning “pre-sleep behaviors myths,” sleep experts focused more on the neurophysiological impacts and provided more evidence-based assessments, with more nuanced explanations of the causes and effects underlying sleep phenomena and disturbances. Finally, regarding “brain function and sleep myths,” sleep experts gave a more detailed explanation of brain functions, highlighting the complexity of sleep research and the presence of controversial topics and conflicting results. Further details are reported in Table S1 in Multimedia Appendix 3.

## Discussion

### Generative Conversational AI and Sleep-Related Myths

AI-driven platforms and agents, including chatbots, can provide sleep-related information and education, ensuring that a diverse global audience can access valuable and customized resources on sleep health. This tailored guidance can be particularly beneficial for improving sleep quality, in that AI-driven chatbots and digital assistants can provide ongoing support and reminders for healthy sleep habits, promoting consistent behavior change over time. However, information disseminated using digital tools must be accurate and reliable.

According to a few studies, the internet can be a useful platform for enhancing sleep-related health literacy, but can also contribute to spreading misinformation, often including commercial biases and incorrect and misleading content. Robbins et al [32] evaluated the understandability, quality of information, and presence of misinformation in popular YouTube videos about sleep compared to those featuring credible experts. The top YouTube videos on sleep or insomnia and 5 expert-led videos were analyzed for clarity and understanding using established tools. Sleep medicine experts agreed on instances of misinformation and commercial bias, with about 67% (n=14/21) of popular videos showing evidence of commercial bias, unlike the expert videos. Misinformation was more prevalent in popular videos that averaged 8.2 million (SD 2.2) views, significantly higher than the expert videos’ 0.3 million (SD 0.2 million) views. Most YouTube videos were found to have clickbait and be appealing to shorter attention spans, having engaging content, good visual quality, and being highly relatable to viewers. All this highlights the issue of misinformation and bias in widely viewed sleep or insomnia videos on YouTube and other web-based platforms, suggesting the urgent need for combating digital sleep-related misinformation [33].

ChatGPT is anticipated to play a key role in sleep health promotion and education, enhancing public perceptions of the importance of sleep in daily life and its impact on human health. This analysis demonstrates the potential for AI tools like ChatGPT to provide health information, in particular in the arena of sleep medicine. Considering the overall distribution of responses provided by ChatGPT, a high proportion of sleep-related myths (n=17/20, 85% of the statements) was correctly identified as either false or generally false, suggesting that ChatGPT is aligned with scientific evidence. However, the categorization of some statements as “true” or “not necessarily true or false” indicates ChatGPT’s ability to recognize and label scientific items as accurate can be still improved.

In general, ChatGPT has a good, scholarly understanding of several crucial aspects of sleep health, spanning from sleep duration and timing to behaviors during sleep, while it demonstrates some limitations in the field of sleep hygiene, and in the understanding of sleep-related occupational and public health implications.

Moreover, addressing sleep myths involves a nuanced exploration of sleep-related topics: our qualitative analysis on how common misconceptions are clarified by both AI platforms such as ChatGPT and sleep experts shows a good alignment, though some statements are approached from different angles. From a qualitative comparative perspective, ChatGPT tends to provide more pragmatic advice and tips, emphasizing the importance of regular sleep schedules and practices, even if in the context of a certain degree of flexibility in sleep systems, and the impact of individual behaviors on sleep quality. This approach often includes general recommendations based on a broad understanding of sleep science, aiming to correct misunderstandings such as the notion that less sleep can be habitually sufficient or that lying in bed with eyes closed substitutes for genuine sleep. In contrast, sleep experts delve deeper into the medical and physiological specifics, offering a more detailed assessment that considers individual health conditions, genetic predispositions, and the long-term health risks associated with disrupted sleep patterns. They might focus on the precise effects of sleep deprivation on cognitive function, the specific dangers of certain presleep behaviors, or the complex, nonlinear relationship between sleep stages and overall health. A major difference between ChatGPT and sleep experts is that only the latter have mentioned the public and occupational aspects of sleep, while the former has focused more on the individual level. The dialogue between these perspectives can enrich our understanding of sleep, blending practical guidance with in-depth scientific insights to debunk myths and promote healthier sleep practices across diverse populations.

However, as previously mentioned, some errors by ChatGPT in correctly classifying myths as false statements underscore the current limitations of AI: users should be aware of the shortcomings of AI-based tools in interpreting complex, evolving fields like sleep science and sleep health. ChatGPT’s classifications are not definitive statements of truth but rather reflections of current knowledge and interpretations, which are constantly evolving. In summary, the categorizations by ChatGPT provide an interesting insight into how AI tools process and present information on complex health topics such as sleep, emphasizing the importance of contextual understanding and the ongoing development of AI capabilities in health education.

### Implications and Future Directions

ChatGPT’s ability to debunk sleep-related myths has several important implications, both in the field of sleep health and in the context of AI in health care and information dissemination. The ability of ChatGPT to accurately debunk sleep-related myths can significantly contribute to enhancing public health education, including sleep health education. Providing reliable information can help correct widespread misconceptions about sleep, which is vital given the importance of sleep for overall health. ChatGPT can also serve as a tool for supporting health care professionals, helping them to stay abreast of the latest advancements, quickly verify information, and provide evidence-based advice to their patients, potentially improving the quality of sleep health advice given.

Moreover, AI-based platforms such as ChatGPT can make sleep health information more accessible to a broader audience and can offer personalized advice based on individual queries, which is difficult to achieve through traditional health education methods.

This study indicates that AI can be a reliable source of health information. However, it also highlights the need for ongoing evaluation to ensure accuracy, especially in areas with nuanced and complex information, such as sleep health. More in detail, this study suggests that while AI tools such as ChatGPT can be highly effective, they should not replace expert opinion but rather complement it. This is particularly important in complex fields where contextual understanding and professional judgment are crucial. There is a need for continuous learning and updating: AI systems must continuously learn and upgrade their knowledge base to ensure the information they provide stays current with the latest scientific findings and expert consensus.

Moreover, ChatGPT’s ability to identify and correct false information is particularly relevant in an era where misinformation can spread rapidly on the web [34-36]. This capability can play a significant role in public health initiatives. On an individual level, accurate AI-driven advice on sleep health can directly contribute to the prevention of diseases, including sleep-related disorders, which are often linked to chronic diseases such as obesity, diabetes, and cardiovascular issues. By debunking myths and offering personalized guidance on healthy sleep practices, these tools can play a pivotal role in enhancing individual wellness, mental health, and overall quality of life. In the broader context of occupational and public health, provided that the above-mentioned shortcomings of ChatGPT in these fields are properly addressed, the dissemination of reliable sleep-related information *via* AI platforms can aid in the formulation of more informed public health policies and initiatives. By increasing the general population’s understanding of the importance of sleep, these tools can contribute to a reduction in health care costs associated with sleep disorders and their comorbidities. Furthermore, the implications for public safety are significant. Improved sleep health, guided by AI-based tools, can lead to decreased incidences of accidents and errors attributed to sleep deprivation, such as those in high-risk professions (eg, transportation, health care, etc). This would not only enhance the safety of the individuals in these roles but also safeguard the broader community. Thus, the integration of AI in sleep health education and promotion aligns with broader public health and safety goals, offering a proactive approach to mitigating risks associated with poor sleep and promoting a healthier, safer society.

Finally, this study opens the door for similar applications of AI in other areas of health and wellness, suggesting a potential for AI tools to become more integrated into various aspects of health care delivery, provided that ethical and practical considerations in addressing misinformation and biases are taken into full account. As previously mentioned, there is a need to constantly monitor and improve AI systems to prevent the spread of misinformation and reduce biases in the information provided. Further, in the context of digital health tools, ensuring the privacy and security of user data is paramount, especially when personal health information is involved, underscoring the need for regulatory and ethical oversight in the use of AI in health care to ensure that these tools are used responsibly and for the benefit of individual, occupational, and public health.

### Strengths and Limitations

This study has some strengths, including its novelty, methodological rigor, and reproducibility. On the other hand, it suffers from several limitations that should be properly acknowledged: future studies should investigate other AI-based tools, such as Google Bard. Not all digital assistants and chatbots have demonstrated efficacy in improving health- and sleep-related behaviors [37,38]. It should be, indeed, considered that each AI-enhanced platform, being trained on different knowledge bases, has specific technical features and capabilities, and, therefore, some AI-based tools may exhibit lower sleep-related knowledge and literacy, demonstrating less capability of correctly identifying the sleep-related statements as false. As such, this implies that monitoring of the AI system should be tool-specific.

### Conclusions

In the present digital era, the synergy of generative conversational AI and sleep health promotion has the potential to positively impact individual, occupational, and public health by providing easy access to evidence-based information and support. This study’s findings demonstrate the potential of AI tools such as ChatGPT in enhancing public health education, particularly in debunking myths and disseminating accurate information related to sleep health. While promising, it is important to use these tools as supplements to, rather than replacements for, sleep expert opinion and to maintain strict standards of accuracy, privacy, and ethical use.

## Appendix

Multimedia Appendix 1 ChatGPT full replies to sleep-related false myths in terms of true/false.

Multimedia Appendix 2 ChatGPT full replies to sleep-related false myths in terms of rating on the 5-point Likert scale.

Multimedia Appendix 3 Qualitative comparison between ChatGPT’s and sleep experts’ appraisals of the falseness and public health significance of sleep-related false.

Multimedia Appendix 4 Verbatim transcription from the interaction with ChatGPT about the truthfulness or falseness of twenty sleep-related false myths.

## Abbreviations

AI: artificial intelligence
ICC: intraclass correlation coefficient

## Data Availability

All data are available within this paper’s text and in Multimedia Appendices 1-3.
