# Supplementary material for: Assessing the Accuracy of Generative Conversational Artificial Intelligence in Debunking Sleep Health Myths: Mixed Methods Comparative Study With Expert Analysis
Source: JMIR Form Res. 2024 Apr 16;8:e55762. doi: 10.2196/55762 (PMC11061787; doi:10.2196/55762)
Supplement: Multimedia Appendix 2 [file formative_v8i1e55762_app2.docx]

**Multimedia Appendix 2.** ChatGPT full replies to sleep-related false myths in terms of rating on the 5-point Likert scale.

| **Sleep-related “false myths”** | **ChatGPT** |
| --- | --- |
| **Sleep duration** | |
| “Being able to fall asleep “anytime, anywhere” is a sign of a healthy sleep system” | The statement "Being able to fall asleep 'anytime, anywhere' is a sign of a healthy sleep system" would generally be rated towards the higher end of the scale, **likely around a 4 or 5** (indicating it is mostly to extremely false). This belief is a common misconception. In a healthy sleep system, the ability to fall asleep is usually governed by a consistent circadian rhythm and a balanced sleep-wake homeostasis. Falling asleep too easily or in inappropriate situations can actually be a sign of sleep deprivation or disorders like narcolepsy. A well-regulated sleep system typically means being able to fall asleep within a reasonable time frame at night and maintaining wakefulness throughout the day. |
| “Many adults need only 5 or less hours of sleep for general health” | The statement "Many adults need only 5 or less hours of sleep for general health" would also be rated towards the higher end of the scale, **likely around a 4 or 5** (indicating it is mostly to extremely false). The vast majority of adults require more than 5 hours of sleep per night for optimal health and functioning. The National Sleep Foundation and numerous sleep studies suggest that most adults need between 7 to 9 hours of sleep per night for optimal health, performance, and safety. Sleeping less than 7 hours on a regular basis is associated with adverse health outcomes, including weight gain, obesity, diabetes, cardiovascular disease, stroke, depression, and a higher risk of death. There are very few individuals, often called "short sleepers," who can maintain their health and functioning with fewer than 6 hours of sleep, but this is considered a genetic rarity rather than the norm. |
| “Your brain and body can learn to function just as well with less sleep” | The statement "Your brain and body can learn to function just as well with less sleep" would be rated as mostly false, **around a 4 on the scale**. This is a common misconception about sleep. While the human body can adapt to certain conditions, including less sleep, this adaptation does not equate to optimal functioning. Chronic sleep deprivation, even if one gets accustomed to it, can have significant negative effects on cognitive function, physical health, mood, and overall well-being. Sleep deprivation impairs attention, alertness, concentration, reasoning, and problem-solving. It can also contribute to various health issues, such as an increased risk of heart disease, kidney disease, high blood pressure, diabetes, and stroke. The body needs adequate sleep to repair itself, consolidate memory, and perform other crucial functions. In essence, while someone might feel they are adapting to less sleep, their performance and health are likely not as optimal as they would be with adequate sleep. |
| “Adults sleep more as they get older” | The statement "Adults sleep more as they get older" would be rated as mostly false, **around a 4 on the scale**. This is a common misconception about sleep patterns in older adults. In reality, as people age, they often experience changes in their sleep patterns, such as becoming sleepier earlier in the evening and waking up earlier in the morning. However, the total amount of sleep they require typically does not increase. In fact, older adults often find it harder to stay asleep for long periods and might experience a decrease in the deep, restorative stages of sleep. Many older adults tend to sleep less than younger adults, not because they need less sleep, but because they often have a harder time falling asleep or staying asleep. This can be due to various factors, including health conditions, medications they are taking, and changes in their internal body clocks. It's important to note that the need for sleep does not decrease with age; older adults still require about 7-8 hours of sleep per night for optimal health and functioning, even though they might find it harder to achieve this amount. |
| “If you can get it, more sleep is always better” | The statement "If you can get it, more sleep is always better" would likely be rated **around a 3 on the scale**, indicating a somewhat false belief. While adequate sleep is crucial for health and well-being, there is such a thing as too much sleep, and it's not necessarily beneficial. Most adults need between 7 to 9 hours of sleep per night for optimal functioning. Sleeping more than this recommended amount on a regular basis can sometimes be associated with health problems. Long sleep durations have been linked to a higher risk of heart disease, diabetes, and depression. Additionally, excessive sleep can be a sign of underlying health issues, such as sleep apnea or other sleep disorders. It's important to find a balance and to listen to your body. Getting the right amount of sleep is more beneficial than simply getting more sleep. Quality of sleep is also crucial. It's not just about the quantity of sleep but also about how restful and undisturbed that sleep is |
| “One night of sleep deprivation will have lasting negative health consequences” | The statement "One night of sleep deprivation will have lasting negative health consequences" would generally be rated as less false, perhaps **around a 2 on the scale**. This is because the impact of a single night of sleep deprivation, while not beneficial, is typically not severe enough to cause lasting negative health consequences for most people. One night of sleep deprivation can lead to immediate but usually temporary effects such as irritability, cognitive impairment, increased stress response, and reduced motor skills. However, these effects are typically short-lived and can often be reversed with a good night's sleep. The real health concerns arise with chronic sleep deprivation, not isolated incidents. Consistently poor sleep over an extended period can lead to more serious health issues, such as an increased risk of obesity, diabetes, cardiovascular disease, and decreased immune function. It's important to distinguish between occasional sleep loss and chronic sleep deprivation. While it's not ideal to miss a night of sleep, for most people, it won't have lasting negative health consequences if their normal sleep pattern is generally healthy. |
| **Sleep timing** | |
| “In terms of your health, it does not matter what time of day you sleep” | The statement "In terms of your health, it does not matter what time of day you sleep" would be rated as mostly false, **around a 4 on the scale**. The timing of sleep does have an impact on health due to the body's circadian rhythms. Circadian rhythms are 24-hour cycles that are part of the body's internal clock, running in the background to carry out essential functions and processes. One of the most important and well-known circadian rhythms is the sleep-wake cycle. Disturbances in these rhythms, such as sleeping at times that are out of sync with the body's natural clock, can have negative effects on health. Sleeping during the day and staying awake at night can disrupt the body's natural rhythm. This disruption can lead to various health issues, including sleep disorders, metabolic problems, cardiovascular disease, obesity, and mood disturbances. Night shift workers, for example, often face these challenges. The optimal time for restorative sleep is generally at night, particularly during the hours of 10 PM and 2 AM, when the body is naturally programmed for sleep. While napping during the day can be beneficial in certain circumstances, the main restorative sleep should ideally occur at night to align with the body's natural circadian rhythms. |
| **Behaviors during sleep** | |
| “Lying in bed with your eyes closed is almost as good as sleeping” | The statement "Lying in bed with your eyes closed is almost as good as sleeping" would be rated as mostly false, **around a 4 on the scale**. While resting with your eyes closed can provide some level of relaxation and rest for the body, it is not nearly as beneficial as actual sleep. Sleep involves various complex processes that are essential for physical and mental health. These processes include muscle repair, memory consolidation, and the release of hormones that regulate growth and appetite. During sleep, the brain goes through specific stages, including REM (rapid eye movement) and non-REM sleep, each playing a critical role in overall health and well-being. Resting quietly with your eyes closed can help reduce stress and fatigue and can be a beneficial way to relax. However, it does not provide the same restorative benefits as sleep. The brain and body engage in numerous vital activities during sleep that cannot be replicated by simply resting. In summary, while resting with your eyes closed is better than no rest at all, it is not a substitute for sleep in terms of the benefits it provides to the body and mind. |
| “If you have difficulty falling asleep, it is best to stay in bed and try to fall back to sleep” | The statement "If you have difficulty falling asleep, it is best to stay in bed and try to fall back to sleep" would be rated as somewhat false, **around a 3 on the scale**. This advice is partially true but needs clarification. While it might seem logical to stay in bed and try to sleep, spending prolonged periods awake in bed can actually lead to a negative association between the bed and sleep. If you're unable to fall asleep within about 20 minutes, sleep experts often recommend getting out of bed and engaging in a quiet, relaxing activity in another room. This could be something like reading or listening to soothing music, avoiding bright lights and screens. The idea is to return to bed only when you feel sleepy. This approach can help prevent the frustration and anxiety associated with lying in bed awake, which can make it even harder to fall asleep. Creating a strong mental association between the bed and sleep is important for long-term sleep health. However, it's also important to maintain a regular sleep schedule. If you frequently find yourself awake at night, it's advisable to consult with a healthcare provider to address any underlying issues. |
| “Although annoying for bed partners, loud snoring is mostly harmless” | The statement "Although annoying for bed partners, loud snoring is mostly harmless" would be rated as somewhat false, **around a 3 on the scale**. While snoring can be harmless for some individuals, it can also be a sign of more serious health issues for others. Loud snoring can be a symptom of obstructive sleep apnea (OSA), a potentially serious sleep disorder. OSA is characterized by repeated stops and starts in breathing during sleep. People with sleep apnea often snore loudly and experience periods during sleep where they stop breathing, which can lead to frequent awakenings and reduced sleep quality. Untreated sleep apnea can lead to a variety of health problems, including daytime fatigue, cardiovascular issues, high blood pressure, stroke, and diabetes. It's important for individuals who snore loudly, especially those who experience other symptoms of sleep apnea (like daytime sleepiness, morning headaches, or observed pauses in breathing during sleep), to consult with a healthcare professional for proper evaluation and treatment. In cases where loud snoring is not associated with sleep apnea or other health conditions, it may be more of a nuisance than a medical concern. However, the distinction should be made through appropriate medical evaluation. |
| “A sound sleeper rarely moves at night” | The statement "A sound sleeper rarely moves at night" would be rated as somewhat false, **around a 3 on the scale**. It's a common misconception that minimal movement during sleep is an indicator of sound or high-quality sleep. In reality, it's normal and healthy to move during sleep. Most people change positions several times during the night. These movements can help relieve pressure on the skin, prevent discomfort, and avoid circulatory problems. Movement during sleep is also a part of the normal sleep cycle, especially during lighter stages of sleep. However, excessive movement during sleep, especially if it involves more dramatic actions like thrashing or kicking, could be indicative of sleep disorders like restless legs syndrome (RLS) or periodic limb movement disorder (PLMD). These conditions can disrupt sleep and might require medical attention. On the other hand, extremely limited movement during sleep could also be a concern, particularly in the context of certain health conditions or sleep disorders. Overall, while being a sound sleeper might involve some degree of stillness, typical movement during sleep is both normal and necessary for physical comfort and health. |
| **Daytime Behaviors that Relate to Sleep** | |
| “Hitting the snooze when you wake up is better than getting up when the alarm first goes off” | The statement "Hitting the snooze when you wake up is better than getting up when the alarm first goes off" would be rated as mostly false, **around a 4 on the scale**. Hitting the snooze button and going back to sleep for a few more minutes often does more harm than good in terms of sleep quality and overall alertness. When you hit the snooze button and fall back asleep, you are likely to start a new sleep cycle that you won't be able to complete. Waking up in the middle of a sleep cycle can leave you feeling groggier and less alert than if you had gotten up with the initial alarm. This phenomenon is known as sleep inertia, and it can negatively impact mood, cognitive functions, and performance, especially shortly after waking. Furthermore, fragmenting your morning sleep with multiple snooze interruptions can disrupt the natural sleep pattern, leading to a feeling of insufficient rest, even if the total sleep time is adequate. It's generally better for sleep quality and morning alertness to set the alarm for the time you actually need to wake up and get out of bed when it goes off. Establishing a consistent wake-up time and a regular sleep schedule can also help improve sleep quality and overall health. |
| “If you are having difficulties sleeping, taking a nap in the afternoon is a good way to get adequate sleep” | The statement "If you are having difficulties sleeping, taking a nap in the afternoon is a good way to get adequate sleep" would be rated as somewhat true, **around a 2 on the scale**. Napping can be a double-edged sword when it comes to managing sleep difficulties. On one hand, short naps, especially those that are around 20 minutes, can help improve mood, alertness, and performance, particularly if you are experiencing sleep deprivation. Napping for a short duration can provide a boost in energy and cognitive function without leading to sleep inertia, the grogginess sometimes felt after waking from a longer nap. However, on the other hand, long or late afternoon naps can interfere with nighttime sleep, especially for individuals with existing sleep difficulties. Napping too long or too late in the day can make it harder to fall asleep at night, thus perpetuating the cycle of sleep difficulties. For people with insomnia or other sleep disorders, napping can sometimes worsen the problem. Therefore, while a short nap can be beneficial for someone who is sleep-deprived, it's important to be cautious about the duration and timing of the nap to ensure it doesn't negatively impact nighttime sleep. For those with chronic sleep difficulties, it's often recommended to avoid napping and instead focus on improving nighttime sleep habits and routines. |
| **Pre-Sleep Behaviors** | |
| “Alcohol before bed will improve your sleep” | The statement "Alcohol before bed will improve your sleep" would be rated as mostly false, **around a 4 on the scale**. While alcohol can make you feel sleepy and might help you fall asleep faster, it actually impairs the quality of your sleep. Alcohol disrupts the sleep cycle and can interfere with REM (rapid eye movement) sleep, which is the most restorative phase of sleep. As the alcohol wears off during the night, you're more likely to experience disturbances in your sleep, leading to a less restful night overall. Additionally, alcohol can exacerbate breathing problems during sleep, including snoring and obstructive sleep apnea. Moreover, relying on alcohol to fall asleep can lead to a dependency, where you might find it increasingly difficult to fall asleep without it. This can create a cycle of poor sleep habits. In summary, while alcohol might seem to help with falling asleep, it ultimately leads to poorer quality sleep and can have negative effects on your overall sleep patterns and health. |
| “For sleeping, it is better to have a warmer bedroom than a cooler bedroom” | The statement "For sleeping, it is better to have a warmer bedroom than a cooler bedroom" would be rated as mostly false, **around a 4 on the scale**. Generally, a cooler bedroom is considered more conducive to good sleep than a warmer one. The body's core temperature naturally drops as you prepare to sleep, and a cooler environment can facilitate this process. Most sleep experts recommend a bedroom temperature between 60 to 67 degrees Fahrenheit (15.6 to 19.4 degrees Celsius) for optimal sleep. This cooler temperature can help signal to your body that it's time to sleep, and can aid in falling asleep more quickly and sleeping more soundly. A room that's too warm can lead to discomfort, restlessness, and difficulty falling asleep or staying asleep. It can also disrupt the quality of REM (rapid eye movement) sleep, an important phase of the sleep cycle. However, personal preferences can vary, and some people may find slightly different temperatures more comfortable. The key is to find a temperature that feels comfortable for sleeping and helps maintain uninterrupted sleep throughout the night. |
| “Boredom can make you sleepy even if you got adequate sleep before” | The statement "Boredom can make you sleepy even if you got adequate sleep before" would be rated as mostly true, **around a 2 on the scale**. Boredom can indeed induce feelings of sleepiness or fatigue, even if you've had enough sleep. When you're bored, you're not engaged or stimulated, which can lead to a decrease in alertness. This reduction in mental engagement can signal the brain that it's time to rest, leading to feelings of drowsiness. This phenomenon is more about the brain's response to a lack of stimulation than actual physical or sleep-related fatigue. It's also worth noting that feeling sleepy in response to boredom doesn't necessarily mean you're sleep-deprived or haven't had adequate sleep. It's more about the brain's current state and its need for stimulation or engagement. When you switch to a more engaging activity, you often find that the sleepiness dissipates. However, if you consistently feel sleepy in situations where you're not mentally stimulated, regardless of how much sleep you've had, it could also be a sign of underlying issues like sleep quality problems or a sleep disorder, and it might be worth discussing with a healthcare provider. |
| “Watching television in bed is a good way to relax before sleep” | The statement "Watching television in bed is a good way to relax before sleep" would be rated as somewhat false, **around a 3 on the scale**. While many people do find watching television to be a relaxing activity before bed, it's not necessarily conducive to promoting good sleep. The main issue with watching TV before bed is the exposure to blue light emitted by the screen. Blue light can interfere with the body's production of melatonin, a hormone that regulates sleep-wake cycles. Exposure to blue light in the evening can trick your brain into thinking it's still daytime, which can make it harder to fall asleep and can disrupt sleep quality. Additionally, engaging content on TV can be mentally stimulating rather than relaxing, potentially increasing alertness and making it more difficult to wind down and fall asleep. For better sleep hygiene, experts often recommend creating a relaxing bedtime routine that avoids screens for at least 30 minutes to an hour before bed. This could include reading a book, taking a warm bath, or practicing relaxation techniques such as meditation or gentle stretching. However, individual responses can vary, and some people might find that watching television helps them unwind effectively. The key is to be mindful of how this habit affects your personal sleep quality. |
| “Exercising within 4 hours of bedtime will disturb your sleep” | The statement "Exercising within 4 hours of bedtime will disturb your sleep" would be rated as somewhat false, **around a 3 on the scale**. The impact of evening exercise on sleep varies from person to person. For some individuals, engaging in vigorous exercise too close to bedtime can be stimulating and may lead to difficulties falling asleep. Exercise raises your heart rate and body temperature, and it can increase alertness, all of which might interfere with the body's natural process of winding down for sleep. However, for others, exercising in the evening does not negatively impact their sleep, and it may even improve sleep quality. Some people may find that physical activity in the evening helps them relax and feel more tired, facilitating better sleep. The effect of exercise on sleep depends on various factors, including an individual's personal fitness level, the type and intensity of the exercise, and their unique circadian rhythms. It's often recommended to pay attention to how evening exercise affects your own sleep and adjust accordingly. If you find that vigorous exercise in the evening disrupts your sleep, you might try switching to more gentle or relaxing activities, like yoga or a leisurely walk. Conversely, if you notice no negative impact, you can continue with your routine. |
| **Brain Function and Sleep** | |
| “During sleep, the brain is not active” | The statement "During sleep, the brain is not active" would be rated as extremely false, **around a 5 on the scale**. This is a common misconception about sleep. In reality, the brain is highly active during sleep, engaging in a variety of important processes. Sleep involves several stages, including REM (rapid eye movement) and non-REM sleep, each characterized by different patterns of brain activity. During REM sleep, the brain is often as active as it is when you are awake. This stage of sleep is associated with vivid dreams, and it plays a crucial role in processes like memory consolidation and emotional regulation. Non-REM sleep, which includes deep sleep stages, is also characterized by significant brain activity. During these stages, the brain is involved in various restorative processes, such as the consolidation of memories, learning, and the clearing of toxins from the brain. Additionally, sleep is essential for cognitive functions such as learning, memory, and problem-solving. Far from being inactive, the brain uses sleep to process information, consolidate memories, and restore its energy for the next day. In summary, the brain is far from inactive during sleep; rather, it's engaged in critical activities essential for overall health and well-being. |
| “Remembering your dreams is a sign of a good night’s sleep” | The statement "Remembering your dreams is a sign of a good night’s sleep" would be rated as somewhat false, **around a 3 on the scale**. Remembering dreams is not a reliable indicator of the quality of sleep. Dream recall varies greatly among individuals and doesn't necessarily correlate with the quality of sleep. People often remember dreams that occur during REM (rapid eye movement) sleep, especially if they wake up during or immediately after this phase. This can happen after a night of good sleep, but it can also occur during fragmented sleep or due to disturbances. Good quality sleep is typically characterized by factors like the appropriate duration of sleep for the individual's age and needs, the ability to fall asleep within a reasonable amount of time, sleeping through the night with minimal disturbances, and waking up feeling rested. Dream recall is more about how sleep is structured and when you wake up relative to your sleep cycles. Therefore, while remembering dreams can be a part of normal sleep experiences, it's not a definitive sign of either good or poor sleep quality. |
