# Supplementary material for: Assessing the Accuracy of Generative Conversational Artificial Intelligence in Debunking Sleep Health Myths: Mixed Methods Comparative Study With Expert Analysis
Source: JMIR Form Res. 2024 Apr 16;8:e55762. doi: 10.2196/55762 (PMC11061787; doi:10.2196/55762)
Supplement: Multimedia Appendix 3 [file formative_v8i1e55762_app3.docx]

**Table S1.** Qualitative comparison between ChatGPT’s and sleep experts’ appraisals of the falseness and public health significance of sleep-related false myths, encompassing a range of 6 different topics (sleep duration, sleep timing, behaviors during sleep, daytime behaviors that relate to sleep, presleep behaviors, and brain function and sleep).

| **Sleep-related “false myths”** | **Similarities** | **Differences** |
| --- | --- | --- |
| **Sleep duration** | | |
| “Being able to fall asleep ‘anytime, anywhere’ is a sign of a healthy sleep system” | Misconception clarification, association with sleep disorders and conditions such as sleep deprivation, and individual (clinical) health implications | Practical advice and emphasis on flexibility in sleep systems, regular sleep practices and hygiene, circadian rhythm, and interindividual variability (ChatGPT) *versus* medical recommendation and focus on individual specific conditions and risks and impact on public health (sleep experts) |
| “Many adults need only 5 or less hours of sleep for general health” | Optimal sleep duration, recognition of short sleepers, acknowledgment of variability, health consequences | Practical advice (ChatGPT) *versus* a more detailed assessment with a focus on genetic predisposition and mention of specific health risks (sleep experts) |
| “Your brain and body can learn to function just as well with less sleep” | Refutation of the statement, adaptation or optimal functioning, long-term health risks | Practical advice (ChatGPT) *versus* detailed appraisal with a focus on the biological and physiological basis of sleep, the subjective-objective sleep discrepancy, and the night-shift workers (sleep experts) |
| “Adults sleep more as they get older” | Changes in sleep patterns, decrease in sleep quantity, duration, and quality, recognition of health factors | Focus on sleep architecture and daytime napping (ChatGPT) *versus* focus on sleep needs versus sleep requirements (sleep experts) |
| “If you can get it, more sleep is always better” | Optimal sleep range, need for balance, indicator of underlying issues, and health risks associated with excessive sleep | General advice (ChatGPT) *versus* detailed appraisal with a focus on specificity of risks, distinction between causal and correlational links, definition of long sleep, performance outcomes, and lack of well-established experimental evidence (sleep experts) |
| “One night of sleep deprivation will have lasting negative health consequences” | Temporary effects, importance of recovery sleep, short-term adverse effects | General advice (ChatGPT) *versus* specificity of effects, long-term perspective, cognitive and performance recovery (sleep experts) |
| **Sleep timing** | | |
| “In terms of your health, it does not matter what time of day you sleep” | Importance of circadian rhythm, adverse effects of off-normal sleep timings, sleep quality and timing, night shift work | More focus on biological and physiological aspects (ChatGPT) *versus* focus on physiopathological aspects (sleep experts) |
| **Behaviors during sleep** | | |
| “Lying in bed with your eyes closed is almost as good as sleeping” | Myth refutation, importance of sleep stages, brain activity, health implications | General advice (ChatGPT) *versus* detailed, technical assessment with a focus on specific physiological processes (sleep experts) |
| “If you have difficulty falling asleep, it is best to stay in bed and try to fall back to sleep” | Avoiding counterproductive behaviors, recommendation of relaxing activities | Tips and pragmatic advice (ChatGPT) *versus* a more detailed, technical appraisal with a focus on stimulus control therapy (sleep experts) |
| “Although annoying for bed partners, loud snoring is mostly harmless” | Recognition of potential severity, link to health complications, advisory for medical consultations | Generalized advice (ChatGPT) *versus* a more detailed and cautionary assessment with a focus on specific health risks, empirical evidence, snoring regardless of OSA (sleep experts) |
| “A sound sleeper rarely moves at night” | Normalcy of movement while sleeping, variability across lifespan, movement not necessarily indicative of poor sleep | Focus on movement during the different sleep stages, excessive movement and disorders (ChatGPT) *versus* focus on age-related effects on movement while sleeping (sleep experts) |
| **Daytime Behaviors that Relate to Sleep** | | |
| “Hitting the snooze when you wake up is better than getting up when the alarm first goes off” | Negative impact of snoozing and recommendation against it | Pragmatic advice (ChatGPT) *versus* focus on the scarcity of scholarly research (sleep experts) |
| “If you are having difficulties sleeping, taking a nap in the afternoon is a good way to get adequate sleep” | Conditional benefits of napping, concerns for individuals with sleep disorders | Cautionary tone (ChatGPT) *versus* focus on cultural and habitual aspects, adverse health outcomes (sleep experts) |
| **Presleep Behaviors** | | |
| “Alcohol before bed will improve your sleep” | REM sleep disruption, increased wakefulness, impact on sleep disorders | Focus on diuretic effect (ChatGPT) *versus* focus on neurophysiological effects, including sleep latency (sleep experts) |
| “For sleeping, it is better to have a warmer bedroom than a cooler bedroom” | Preference for cooler temperatures, discomfort in warm condition | Practical advice with a focus on bedroom temperature and quality of REM sleep (ChatGPT) *versus* a more detailed, evidence-based technical assessment (sleep experts) |
| “Boredom can make you sleepy even if you got adequate sleep before” | Boredom and sleepiness connection, conditions and factors underlying sleepiness, engagement levels | Focus on direct causal links (ChatGPT) *versus* a more nuanced explanatory theory in terms of causes, effects, and impacts (sleep experts) |
| “Watching television in bed is a good way to relax before sleep” | Sleep disruption, mental stimulation, acknowledgment of individual variability | Practical tips and focus on blue light concerns (ChatGPT) *versus* a more detailed assessment with a focus on presleep routines and epidemiological data (sleep experts) |
| “Exercising within 4 hours of bedtime will disturb your sleep” | Individual variability, potential benefits of exercise on sleep | Focus on sleep latency and REM sleep (ChatGPT) *versus* focus on lack of evidence and contrasting findings reported in the scholarly literature (sleep experts) |
| **Brain Function and Sleep** | | |
| “During sleep, the brain is not active” | Brain activity in REM sleep, restorative function, brain wave patterns | Refutation with a focus on some physiological aspects like clearance of neurotoxic waste (sleep experts) |
| “Remembering your dreams is a sign of a good night’s sleep” | Association with REM sleep | Focus on the lack of association between dream recall and sleep quality, other possible hypotheses such as sleep fragmentation and disruption (ChatGPT) *versus* focus on the complexity of dream research (sleep experts) |
