# Supplementary material for: Assessing the Accuracy of Generative Conversational Artificial Intelligence in Debunking Sleep Health Myths: Mixed Methods Comparative Study With Expert Analysis
Source: JMIR Form Res. 2024 Apr 16;8:e55762. doi: 10.2196/55762 (PMC11061787; doi:10.2196/55762)
Supplement: Multimedia Appendix 4 [file formative_v8i1e55762_app4.docx]

**Multimedia Appendix 4.** Verbatim transcription from the interaction with ChatGPT about the truthfulness or falseness of twenty sleep-related false myths.

NLB: Is the statement “Being able to fall asleep “anytime, anywhere” is a sign of a healthy sleep system” true or false?

ChatGPT: The statement that being able to fall asleep "anytime, anywhere" is a sign of a healthy sleep system is generally false. While it might seem like a convenient ability, it can actually be indicative of sleep deprivation or a sleep disorder. In a healthy sleep system, an individual typically follows a regular sleep pattern, falling asleep easily at night and staying awake and alert during the day. The ability to fall asleep quickly in any setting, especially during times or in places not meant for sleeping, can suggest that the person is not getting enough quality sleep at night. This could be due to various factors like insomnia, sleep apnea, or other sleep disorders. However, this is not a one-size-fits-all answer. Some people might naturally have a more flexible sleep system, allowing them to nap easily without indicating any underlying problems. But for most, especially if this ability is coupled with feelings of fatigue or sleepiness during the day, it may be worth consulting a healthcare professional to rule out sleep disorders or other health issues.

NLB: On a 5-point Likert scale, from 1 or “not at all false” to 5 or “extremely false”, how would you rate this statement?

ChatGPT: The statement "Being able to fall asleep 'anytime, anywhere' is a sign of a healthy sleep system" would generally be rated towards the higher end of the scale, likely around a 4 or 5 (indicating it is mostly to extremely false). This belief is a common misconception. In a healthy sleep system, the ability to fall asleep is usually governed by a consistent circadian rhythm and a balanced sleep-wake homeostasis. Falling asleep too easily or in inappropriate situations can actually be a sign of sleep deprivation or disorders like narcolepsy. A well-regulated sleep system typically means being able to fall asleep within a reasonable time frame at night and maintaining wakefulness throughout the day.

NLB: Is the statement “Many adults need only 5 or less hours of sleep for general health” true or false?

ChatGPT: The statement that many adults need only 5 or less hours of sleep for general health is false. The majority of adults require between 7 and 9 hours of sleep per night for optimal health and well-being. While sleep needs can vary slightly from person to person, consistently getting less than the recommended amount of sleep can lead to negative health consequences. Chronic sleep deprivation has been linked to various issues, including an increased risk of heart disease, obesity, diabetes, impaired cognitive function, and reduced immune function. There is a small percentage of the population, often referred to as "short sleepers," who can function well on fewer than 6 hours of sleep per night due to a genetic predisposition. However, this is quite rare and does not apply to the majority of adults. Most adults should aim for at least 7 hours of sleep per night for good health and well-being. If someone consistently finds themselves needing less than 5 hours of sleep and feeling well-rested, it might be an anomaly, but it's not the norm for general health.

NLB: On a 5-point Likert scale, from 1 or “not at all false” to 5 or “extremely false”, how would you rate this statement?

ChatGPT: The statement "Many adults need only 5 or less hours of sleep for general health" would also be rated towards the higher end of the scale, likely around a 4 or 5 (indicating it is mostly to extremely false). The vast majority of adults require more than 5 hours of sleep per night for optimal health and functioning. The National Sleep Foundation and numerous sleep studies suggest that most adults need between 7 to 9 hours of sleep per night for optimal health, performance, and safety. Sleeping less than 7 hours on a regular basis is associated with adverse health outcomes, including weight gain, obesity, diabetes, cardiovascular disease, stroke, depression, and a higher risk of death. There are very few individuals, often called "short sleepers," who can maintain their health and functioning with fewer than 6 hours of sleep, but this is considered a genetic rarity rather than the norm.

NLB: Is the statement “Your brain and body can learn to function just as well with less sleep” true or false?

ChatGPT: The statement that your brain and body can learn to function just as well with less sleep is false. While people can adapt to a certain degree of sleep deprivation and may feel like they are functioning adequately, this doesn't mean their brain and body are performing optimally. Chronic sleep deprivation, even if it's just an hour or two less than the recommended amount, can lead to significant impairments in cognitive functions such as memory, attention, and decision-making. Additionally, insufficient sleep can have negative effects on physical health, including increased risks for obesity, heart disease, diabetes, and a weakened immune system. The body's need for sleep is a biological requirement, much like the need for food or water. While short-term adaptations might mask the immediate effects of sleep loss, the long-term consequences can be detrimental to health and well-being. Regular, sufficient sleep is crucial for maintaining cognitive function, physical health, and overall quality of life.

NLB: On a 5-point Likert scale, from 1 or “not at all false” to 5 or “extremely false”, how would you rate this statement?

ChatGPT: The statement "Your brain and body can learn to function just as well with less sleep" would be rated as mostly false, around a 4 on the scale. This is a common misconception about sleep. While the human body can adapt to certain conditions, including less sleep, this adaptation does not equate to optimal functioning. Chronic sleep deprivation, even if one gets accustomed to it, can have significant negative effects on cognitive function, physical health, mood, and overall well-being. Sleep deprivation impairs attention, alertness, concentration, reasoning, and problem-solving. It can also contribute to various health issues, such as an increased risk of heart disease, kidney disease, high blood pressure, diabetes, and stroke. The body needs adequate sleep to repair itself, consolidate memory, and perform other crucial functions. In essence, while someone might feel they are adapting to less sleep, their performance and health are likely not as optimal as they would be with adequate sleep.

NLB: Is the statement “Adults sleep more as they get older” true or false?

ChatGPT: The statement that adults sleep more as they get older is generally false. In reality, as adults age, they often experience changes in their sleep patterns, including a decrease in the duration and quality of sleep. Elderly adults may find: Shorter Sleep Duration: Older adults often sleep less than younger adults. This is partly due to changes in the body's internal clock and partly due to increased prevalence of health issues and sleep disturbances. More Fragmented Sleep: Sleep tends to become more fragmented with age, meaning older adults might wake up more often during the night. Early Bed and Rise Times: There's a tendency for older adults to go to bed earlier and wake up earlier than they did in their younger years. Increased Daytime Napping: Older adults may nap more during the day, but this does not necessarily compensate for decreased nighttime sleep. Changes in Sleep Architecture: There is less time spent in deep, restorative stages of sleep as one ages. It's important to note that while these changes are common, they are not necessarily a normal part of aging. Often, sleep disturbances in older adults are related to medical and psychiatric illnesses, medications, or other sleep disorders. Despite these changes, the need for restful sleep remains important throughout the lifespan for maintaining health and well-being.

NLB: On a 5-point Likert scale, from 1 or “not at all false” to 5 or “extremely false”, how would you rate this statement?

ChatGPT: The statement "Adults sleep more as they get older" would be rated as mostly false, around a 4 on the scale. This is a common misconception about sleep patterns in older adults. In reality, as people age, they often experience changes in their sleep patterns, such as becoming sleepier earlier in the evening and waking up earlier in the morning. However, the total amount of sleep they require typically does not increase. In fact, older adults often find it harder to stay asleep for long periods and might experience a decrease in the deep, restorative stages of sleep. Many older adults tend to sleep less than younger adults, not because they need less sleep, but because they often have a harder time falling asleep or staying asleep. This can be due to various factors, including health conditions, medications they are taking, and changes in their internal body clocks. It's important to note that the need for sleep does not decrease with age; older adults still require about 7-8 hours of sleep per night for optimal health and functioning, even though they might find it harder to achieve this amount.

NLB: Is the statement “If you can get it, more sleep is always better” true or false?

ChatGPT: The statement that more sleep is always better is false. While adequate sleep is essential for health and well-being, there is such a thing as too much sleep, which can also be problematic. The optimal amount of sleep for most adults is between 7 to 9 hours per night. Regularly sleeping more than this recommended range may be associated with certain health risks, such as: Increased Risk of Heart Disease and Stroke: Some studies have shown a correlation between excessive sleep and an increased risk of cardiovascular problems. Higher Risk of Metabolic Issues: Prolonged sleep durations have been linked with a higher risk of obesity, insulin resistance, and type 2 diabetes. Mental Health Concerns: Oversleeping is often linked with mental health issues such as depression and anxiety. Cognitive Impairment: Too much sleep can impact cognitive function, leading to issues like impaired memory and decreased alertness. It is important to note that in some cases, the need for excessive sleep can be a symptom of an underlying health issue, such as a sleep disorder, depression, or other medical conditions. Therefore, consistently sleeping much more than the recommended amount should be discussed with a healthcare provider to determine if there's an underlying cause that needs attention. In summary, like many things in health, balance is key. Getting enough sleep is crucial, but more is not always better.

NLB: On a 5-point Likert scale, from 1 or “not at all false” to 5 or “extremely false”, how would you rate this statement?

ChatGPT: The statement "If you can get it, more sleep is always better" would likely be rated around a 3 on the scale, indicating a somewhat false belief. While adequate sleep is crucial for health and well-being, there is such a thing as too much sleep, and it's not necessarily beneficial. Most adults need between 7 to 9 hours of sleep per night for optimal functioning. Sleeping more than this recommended amount on a regular basis can sometimes be associated with health problems. Long sleep durations have been linked to a higher risk of heart disease, diabetes, and depression. Additionally, excessive sleep can be a sign of underlying health issues, such as sleep apnea or other sleep disorders. It's important to find a balance and to listen to your body. Getting the right amount of sleep is more beneficial than simply getting more sleep. Quality of sleep is also crucial. It's not just about the quantity of sleep but also about how restful and undisturbed that sleep is.

NLB: Is the statement “One night of sleep deprivation will have lasting negative health consequences” true or false?

ChatGPT: The statement that one night of sleep deprivation will have lasting negative health consequences is generally false. While a single night of poor sleep can temporarily affect mood, cognitive function, and physical performance, these effects are usually short-lived and can typically be reversed with subsequent good sleep. The immediate effects of a single night of sleep deprivation might include: Reduced Alertness and Impaired Cognitive Function: This can impact memory, decision-making, and concentration. Mood Changes: Increased irritability, stress, and likelihood of emotional responses. Physical Effects: Changes in appetite, reduced coordination, and increased fatigue. However, these effects are not usually long-lasting and can often be resolved by returning to a normal sleep pattern. It is when sleep deprivation becomes chronic (repeated over time) that there are significant risks for long-term health consequences, such as an increased risk for chronic diseases like heart disease, obesity, diabetes, and mental health disorders. So, while one night of poor sleep is not ideal and can have short-term negative effects, it is unlikely to cause lasting health consequences if it is an isolated incident.

NLB: On a 5-point Likert scale, from 1 or “not at all false” to 5 or “extremely false”, how would you rate this statement?

ChatGPT: The statement "One night of sleep deprivation will have lasting negative health consequences" would generally be rated as less false, perhaps around a 2 on the scale. This is because the impact of a single night of sleep deprivation, while not beneficial, is typically not severe enough to cause lasting negative health consequences for most people. One night of sleep deprivation can lead to immediate but usually temporary effects such as irritability, cognitive impairment, increased stress response, and reduced motor skills. However, these effects are typically short-lived and can often be reversed with a good night's sleep. The real health concerns arise with chronic sleep deprivation, not isolated incidents. Consistently poor sleep over an extended period can lead to more serious health issues, such as an increased risk of obesity, diabetes, cardiovascular disease, and decreased immune function. It's important to distinguish between occasional sleep loss and chronic sleep deprivation. While it's not ideal to miss a night of sleep, for most people, it won't have lasting negative health consequences if their normal sleep pattern is generally healthy.

NLB: Is the statement“In terms of your health, it does not matter what time of day you sleep” true or false?

ChatGPT: The statement that it does not matter what time of day you sleep, in terms of health, is generally false. The timing of sleep does matter due to the body's internal clock, known as the circadian rhythm, which is influenced by environmental cues, especially light. Here's why the timing of sleep is important: Circadian Rhythm: This natural internal process regulates the sleep-wake cycle and repeats roughly every 24 hours. It is aligned not only with the cycle of day and night but also with various physiological processes such as hormone production, brain wave activity, and cell regeneration. Quality of Sleep: The circadian rhythm typically promotes the deepest and most restorative sleep during nighttime hours. Sleeping during the day can lead to less restorative sleep, as it often results in shorter sleep duration and more fragmented sleep. Health Implications: Misalignment of the sleep-wake cycle and circadian rhythms can have adverse health effects. Shift workers, for example, who often sleep during the day and work at night, are at a higher risk for certain health problems, including sleep disorders, metabolic syndrome, cardiovascular diseases, and mood disorders. Natural Light Exposure: Exposure to natural light and darkness at appropriate times is crucial for maintaining a healthy circadian rhythm. Daytime light exposure stimulates wakefulness and alertness, while darkness promotes the secretion of melatonin, which aids in sleep. While some people, such as shift workers, may have no choice but to sleep during the day, it is generally healthier to sleep at night when possible. Aligning sleep with the body's natural circadian rhythm promotes better sleep quality and overall health.

NLB: On a 5-point Likert scale, from 1 or “not at all false” to 5 or “extremely false”, how would you rate this statement?

ChatGPT: The statement "In terms of your health, it does not matter what time of day you sleep" would be rated as mostly false, around a 4 on the scale. The timing of sleep does have an impact on health due to the body's circadian rhythms. Circadian rhythms are 24-hour cycles that are part of the body's internal clock, running in the background to carry out essential functions and processes. One of the most important and well-known circadian rhythms is the sleep-wake cycle. Disturbances in these rhythms, such as sleeping at times that are out of sync with the body's natural clock, can have negative effects on health. Sleeping during the day and staying awake at night can disrupt the body's natural rhythm. This disruption can lead to various health issues, including sleep disorders, metabolic problems, cardiovascular disease, obesity, and mood disturbances. Night shift workers, for example, often face these challenges. The optimal time for restorative sleep is generally at night, particularly during the hours of 10 PM and 2 AM, when the body is naturally programmed for sleep. While napping during the day can be beneficial in certain circumstances, the main restorative sleep should ideally occur at night to align with the body's natural circadian rhythms.

NLB: Is the statement “Lying in bed with your eyes closed is almost as good as sleeping” true or false?

ChatGPT: The statement that lying in bed with your eyes closed is almost as good as sleeping is false. While resting with your eyes closed can be relaxing and may provide some benefits, it does not substitute for the profound restorative effects of actual sleep. Here's why sleep is significantly different and more beneficial than just lying in bed with eyes closed: Sleep Stages: Sleep involves several stages, including REM (rapid eye movement) and various stages of non-REM sleep, each of which serves critical restorative functions for the brain and body. These stages facilitate processes like memory consolidation, muscle repair, and hormone regulation. Brain Activity: During sleep, the brain undergoes specific patterns of activity that are essential for cognitive functions such as learning, memory, and problem-solving. Resting with eyes closed does not replicate these complex brain activities. Physical Restoration: Sleep is crucial for physical health; it's when the body repairs tissues, builds bone and muscle, and strengthens the immune system. Simply lying down with eyes closed doesn't provide these same benefits. Mental Health: Adequate sleep is vital for mental health, affecting mood, stress levels, and emotional regulation. Resting without sleeping does not have the same positive impact on mental well-being. Energy Conservation: While resting can help conserve some energy, sleep is more effective in ensuring energy restoration for the next day. Quality of Rest: The quality of rest obtained from sleep, in terms of both mental and physical recovery, is far superior to simply lying down with eyes closed. While it's important to rest and relax, especially if you can't sleep, it's crucial to recognize that rest is not a substitute for sleep. If you're having trouble sleeping, it's better to address the underlying issues with appropriate sleep hygiene practices or seek medical advice.

NLB: On a 5-point Likert scale, from 1 or “not at all false” to 5 or “extremely false”, how would you rate this statement?

ChatGPT: The statement "Lying in bed with your eyes closed is almost as good as sleeping" would be rated as mostly false, around a 4 on the scale. While resting with your eyes closed can provide some level of relaxation and rest for the body, it is not nearly as beneficial as actual sleep. Sleep involves various complex processes that are essential for physical and mental health. These processes include muscle repair, memory consolidation, and the release of hormones that regulate growth and appetite. During sleep, the brain goes through specific stages, including REM (rapid eye movement) and non-REM sleep, each playing a critical role in overall health and well-being. Resting quietly with your eyes closed can help reduce stress and fatigue and can be a beneficial way to relax. However, it does not provide the same restorative benefits as sleep. The brain and body engage in numerous vital activities during sleep that cannot be replicated by simply resting. In summary, while resting with your eyes closed is better than no rest at all, it is not a substitute for sleep in terms of the benefits it provides to the body and mind.

NLB: Is the statement “If you have difficulty falling asleep, it is best to stay in bed and try to fall back to sleep” true or false?

ChatGPT: The statement that if you have difficulty falling asleep, it is best to stay in bed and try to fall back to sleep, is generally considered false according to sleep experts. This advice is based on the principles of sleep hygiene and cognitive behavioral therapy for insomnia (CBT-I). When you can't fall asleep or wake up and can't get back to sleep, staying in bed while awake can lead to a negative association between your bed and wakefulness. Instead, it's often recommended to: Leave the Bed: If you haven't fallen asleep within about 20 minutes (this is an estimate, as it's not suggested to watch the clock), leave your bed and go to another room. Engage in a Calming Activity: Do something relaxing, such as reading or listening to soft music, under low light. Avoid activities that are stimulating or involve screens, as the light from these devices can suppress melatonin production and make it harder to sleep. Return to Bed When Sleepy: Go back to bed when you're feeling sleepy again. Maintain a Regular Schedule: Try to get up at the same time each morning, even if you didn't sleep well. This helps regulate your body's clock and can improve your sleep over time. Relaxation Techniques: Practice relaxation methods like deep breathing, progressive muscle relaxation, or meditation to help your body relax and prepare for sleep. Avoid Clock-Watching: Constantly checking the time can increase stress and make it harder to fall asleep. This approach is part of good sleep hygiene and is aimed at strengthening the bed-sleep connection. If sleep difficulties persist, it may be helpful to consult a healthcare provider or a sleep specialist.

NLB: On a 5-point Likert scale, from 1 or “not at all false” to 5 or “extremely false”, how would you rate this statement?

ChatGPT: The statement "If you have difficulty falling asleep, it is best to stay in bed and try to fall back to sleep" would be rated as somewhat false, around a 3 on the scale. This advice is partially true but needs clarification. While it might seem logical to stay in bed and try to sleep, spending prolonged periods awake in bed can actually lead to a negative association between the bed and sleep. If you're unable to fall asleep within about 20 minutes, sleep experts often recommend getting out of bed and engaging in a quiet, relaxing activity in another room. This could be something like reading or listening to soothing music, avoiding bright lights and screens. The idea is to return to bed only when you feel sleepy. This approach can help prevent the frustration and anxiety associated with lying in bed awake, which can make it even harder to fall asleep. Creating a strong mental association between the bed and sleep is important for long-term sleep health. However, it's also important to maintain a regular sleep schedule. If you frequently find yourself awake at night, it's advisable to consult with a healthcare provider to address any underlying issues.

NLB: Is the statement “Although annoying for bed partners, loud snoring is mostly harmless” true or false?

ChatGPT: The statement that loud snoring is mostly harmless is false. While snoring can be a common and benign issue for some people, it can also be a sign of a more serious health condition, especially if it is loud and habitual. Loud and persistent snoring can be an indicator of obstructive sleep apnea (OSA), a condition where breathing repeatedly stops and starts during sleep. This is due to the relaxation of throat muscles, which causes a temporary blockage of the airway. Sleep apnea can lead to several health problems, including: Interrupted Breathing: Individuals with sleep apnea can experience brief periods during sleep where they stop breathing, which can happen multiple times per night. Reduced Oxygen Levels: These breathing interruptions can lead to lower oxygen levels in the blood, putting strain on the cardiovascular system. Increased Risk of Heart Disease: People with untreated sleep apnea have a higher risk of high blood pressure, heart attack, stroke, and irregular heartbeats. Daytime Fatigue: Sleep apnea can significantly disrupt sleep, leading to excessive daytime sleepiness, which can impair performance at work or school and increase the risk of accidents. Mental Health Issues: Chronic sleep disruption can contribute to depression, anxiety, and cognitive impairment. Other Health Issues: Sleep apnea has been linked with metabolic syndrome, liver problems, and complications with medications and surgery. It's important to note that not everyone who snores has sleep apnea. However, if snoring is loud, frequent, and accompanied by daytime fatigue, gasping or choking sounds during sleep, or pauses in breathing, it is advisable to consult a healthcare provider for evaluation. Early diagnosis and treatment can reduce the risk of health complications and improve the quality of sleep.

NLB: On a 5-point Likert scale, from 1 or “not at all false” to 5 or “extremely false”, how would you rate this statement?

ChatGPT: The statement "Although annoying for bed partners, loud snoring is mostly harmless" would be rated as somewhat false, around a 3 on the scale. While snoring can be harmless for some individuals, it can also be a sign of more serious health issues for others. Loud snoring can be a symptom of obstructive sleep apnea (OSA), a potentially serious sleep disorder. OSA is characterized by repeated stops and starts in breathing during sleep. People with sleep apnea often snore loudly and experience periods during sleep where they stop breathing, which can lead to frequent awakenings and reduced sleep quality. Untreated sleep apnea can lead to a variety of health problems, including daytime fatigue, cardiovascular issues, high blood pressure, stroke, and diabetes. It's important for individuals who snore loudly, especially those who experience other symptoms of sleep apnea (like daytime sleepiness, morning headaches, or observed pauses in breathing during sleep), to consult with a healthcare professional for proper evaluation and treatment. In cases where loud snoring is not associated with sleep apnea or other health conditions, it may be more of a nuisance than a medical concern. However, the distinction should be made through appropriate medical evaluation.

NLB: Is the statement “A sound sleeper rarely moves at night” true or false?

ChatGPT: The statement that a sound sleeper rarely moves at night is false. Movement during sleep is normal and occurs in all people to some extent. In fact, it's quite common and natural to change positions multiple times during the night. Here are some key points about movement during sleep: Sleep Stages and Movement: Sleep is composed of several stages, including REM (rapid eye movement) and non-REM sleep. While REM sleep is characterized by temporary paralysis of most voluntary muscles (which prevents acting out dreams), non-REM sleep, especially the lighter stages, can involve some tossing and turning. Normal Body Functions: Movement in sleep can be a part of the body's normal functioning. It can help relieve pressure and prevent discomfort or bedsores that might occur from staying in one position too long. Not a Disturbance Indicator: Frequent movement during sleep doesn't necessarily indicate a disturbed sleep or a sleep disorder. Some people are just more active in their sleep than others. Excessive Movement: While normal movement is part of healthy sleep, excessive movement could be a sign of a sleep disorder, such as restless legs syndrome or periodic limb movement disorder. If movements are significantly disrupting sleep, it might be worth discussing with a healthcare provider. Individual Variability: There's considerable individual variability in how much people move during sleep, and it doesn't directly correlate with the quality of sleep. In summary, being a sound sleeper does not equate to being motionless throughout the night. Normal movement during sleep is part of the body's natural sleep process.

NLB: On a 5-point Likert scale, from 1 or “not at all false” to 5 or “extremely false”, how would you rate this statement?

ChatGPT: The statement "A sound sleeper rarely moves at night" would be rated as somewhat false, around a 3 on the scale. It's a common misconception that minimal movement during sleep is an indicator of sound or high-quality sleep. In reality, it's normal and healthy to move during sleep. Most people change positions several times during the night. These movements can help relieve pressure on the skin, prevent discomfort, and avoid circulatory problems. Movement during sleep is also a part of the normal sleep cycle, especially during lighter stages of sleep. However, excessive movement during sleep, especially if it involves more dramatic actions like thrashing or kicking, could be indicative of sleep disorders like restless legs syndrome (RLS) or periodic limb movement disorder (PLMD). These conditions can disrupt sleep and might require medical attention. On the other hand, extremely limited movement during sleep could also be a concern, particularly in the context of certain health conditions or sleep disorders. Overall, while being a sound sleeper might involve some degree of stillness, typical movement during sleep is both normal and necessary for physical comfort and health.

NLB: Is the statement “Hitting the snooze when you wake up is better than getting up when the alarm first goes off” true or false?

ChatGPT: The statement that hitting the snooze button when you wake up is better than getting up when the alarm first goes off is generally considered false. Hitting the snooze button can actually disrupt your sleep cycle and may lead to sleep inertia, making you feel groggier and less alert than if you had gotten up with the initial alarm. Here are a few reasons why hitting snooze is not recommended: Fragmented Sleep: Snoozing leads to fragmented sleep, which is of poorer quality compared to continuous sleep. The short bouts of sleep between snoozes are not long enough to reach the deeper, more restorative stages of sleep. Sleep Inertia: Waking up and then going back to sleep can confuse your brain and body, leading to a groggier feeling upon waking. This phenomenon, known as sleep inertia, can affect your alertness and performance for a while after you finally get up. Disrupting the Sleep Cycle: When you fall back asleep after hitting snooze, your body may start a new sleep cycle that it won't have time to finish, resulting in feeling more tired. Mental Health: Regularly disrupting your final sleep stages can have negative impacts on mood and cognitive function. Stress: Waking up multiple times in the morning can also add stress, as you might constantly worry about oversleeping. To promote better sleep and morning habits, it's recommended to set your alarm for the time you actually need to wake up and to get out of bed when it goes off. If you consistently find it hard to wake up, it might be a sign that you need to adjust your bedtime routine or sleep environment. Consistently getting enough sleep and maintaining a regular sleep schedule are key to waking up refreshed.

NLB: On a 5-point Likert scale, from 1 or “not at all false” to 5 or “extremely false”, how would you rate this statement?

ChatGPT: The statement "Hitting the snooze when you wake up is better than getting up when the alarm first goes off" would be rated as mostly false, around a 4 on the scale. Hitting the snooze button and going back to sleep for a few more minutes often does more harm than good in terms of sleep quality and overall alertness. When you hit the snooze button and fall back asleep, you are likely to start a new sleep cycle that you won't be able to complete. Waking up in the middle of a sleep cycle can leave you feeling groggier and less alert than if you had gotten up with the initial alarm. This phenomenon is known as sleep inertia, and it can negatively impact mood, cognitive functions, and performance, especially shortly after waking. Furthermore, fragmenting your morning sleep with multiple snooze interruptions can disrupt the natural sleep pattern, leading to a feeling of insufficient rest, even if the total sleep time is adequate. It's generally better for sleep quality and morning alertness to set the alarm for the time you actually need to wake up and get out of bed when it goes off. Establishing a consistent wake-up time and a regular sleep schedule can also help improve sleep quality and overall health.

NLB: Is the statement “If you are having difficulties sleeping, taking a nap in the afternoon is a good way to get adequate sleep” true or false?

ChatGPT: The statement that taking a nap in the afternoon is a good way to get adequate sleep if you are having difficulties sleeping at night is generally false, especially for individuals experiencing chronic sleep issues. While short naps (about 20-30 minutes) can be beneficial for a quick boost in alertness and well-being, especially for those who are sleep-deprived, they are not a complete substitute for a full night's sleep. Moreover, for people with insomnia or difficulties sleeping at night, napping during the day can actually exacerbate the problem: Sleep Cycle Disruption: Napping, especially for longer periods or later in the day, can interfere with the natural sleep-wake cycle (circadian rhythm). This can make it harder to fall asleep at night. Sleep Pressure: Sleep drive, or the body’s need for sleep, builds up throughout the day. Napping can decrease this sleep pressure, making it more difficult to fall asleep at the usual bedtime. Sleep Pattern Disturbances: Regularly napping during the day can lead to a pattern of fragmented and unrefreshing sleep, further complicating sleep difficulties. For individuals struggling with regular nighttime sleep, it's often more beneficial to focus on strategies that promote better sleep at night, such as: Establishing a consistent sleep schedule. Practicing good sleep hygiene (e.g., keeping the bedroom dark and cool, avoiding caffeine and electronics before bed). Using relaxation techniques to wind down. If naps are necessary due to extreme tiredness, they should be limited to early afternoon and kept short to minimize their impact on nighttime sleep. However, if sleep difficulties persist, it's advisable to consult with a healthcare provider or sleep specialist for personalized advice and possible treatment.

NLB: On a 5-point Likert scale, from 1 or “not at all false” to 5 or “extremely false”, how would you rate this statement?

ChatGPT: The statement "If you are having difficulties sleeping, taking a nap in the afternoon is a good way to get adequate sleep" would be rated as somewhat true, around a 2 on the scale. Napping can be a double-edged sword when it comes to managing sleep difficulties. On one hand, short naps, especially those that are around 20 minutes, can help improve mood, alertness, and performance, particularly if you are experiencing sleep deprivation. Napping for a short duration can provide a boost in energy and cognitive function without leading to sleep inertia, the grogginess sometimes felt after waking from a longer nap. However, on the other hand, long or late afternoon naps can interfere with nighttime sleep, especially for individuals with existing sleep difficulties. Napping too long or too late in the day can make it harder to fall asleep at night, thus perpetuating the cycle of sleep difficulties. For people with insomnia or other sleep disorders, napping can sometimes worsen the problem. Therefore, while a short nap can be beneficial for someone who is sleep-deprived, it's important to be cautious about the duration and timing of the nap to ensure it doesn't negatively impact nighttime sleep. For those with chronic sleep difficulties, it's often recommended to avoid napping and instead focus on improving nighttime sleep habits and routines.

NLB: Is the statement “Alcohol before bed will improve your sleep” true or false?

ChatGPT: The statement that alcohol before bed will improve your sleep is false. While alcohol can make you feel drowsy and may help you fall asleep faster, it negatively impacts the quality of your sleep. Here's how alcohol affects sleep: Disrupts Sleep Cycles: Alcohol can disrupt the sleep cycle, particularly the REM (rapid eye movement) stage, which is important for memory and learning. Drinking before bed can lead to less REM sleep, making sleep less restful and restorative. Increases Wakefulness During the Night: As the body metabolizes alcohol, it can lead to increased wakefulness in the second half of the night, which can disrupt sleep. Exacerbates Breathing Problems: Alcohol relaxes the muscles of the throat, which can worsen snoring and obstructive sleep apnea, leading to disrupted sleep. Causes Frequent Bathroom Trips: Alcohol is a diuretic, meaning it increases urine production, which can lead to more frequent trips to the bathroom during the night. Affects Sleep Quality: Even if alcohol helps you fall asleep, the overall quality of sleep is often poorer, leading to feeling less rested in the morning. While a small amount of alcohol might not significantly disrupt sleep for some people, it's generally advisable to avoid alcohol close to bedtime to ensure better sleep quality. If you're struggling with sleep, consider other relaxation techniques and establish a healthy bedtime routine for better sleep hygiene.

NLB: On a 5-point Likert scale, from 1 or “not at all false” to 5 or “extremely false”, how would you rate this statement?

ChatGPT: The statement "Alcohol before bed will improve your sleep" would be rated as mostly false, around a 4 on the scale. While alcohol can make you feel sleepy and might help you fall asleep faster, it actually impairs the quality of your sleep. Alcohol disrupts the sleep cycle and can interfere with REM (rapid eye movement) sleep, which is the most restorative phase of sleep. As the alcohol wears off during the night, you're more likely to experience disturbances in your sleep, leading to a less restful night overall. Additionally, alcohol can exacerbate breathing problems during sleep, including snoring and obstructive sleep apnea. Moreover, relying on alcohol to fall asleep can lead to a dependency, where you might find it increasingly difficult to fall asleep without it. This can create a cycle of poor sleep habits. In summary, while alcohol might seem to help with falling asleep, it ultimately leads to poorer quality sleep and can have negative effects on your overall sleep patterns and health.

NLB: Is the statement “For sleeping, it is better to have a warmer bedroom than a cooler bedroom” true or false?

ChatGPT: The statement that it is better to have a warmer bedroom than a cooler one for sleeping is false. In general, a cooler bedroom is considered better for sleep. Here's why a cooler environment is often recommended for sleeping: Body Temperature Regulation: Your body naturally drops in temperature as you fall asleep. A cooler room can facilitate this drop, making it easier to fall asleep and stay asleep. Improved Sleep Quality: Many people find they sleep more soundly in a cooler environment. Overheating can disrupt sleep, leading to more awakenings and lighter sleep. Optimal Temperature Range: The ideal room temperature for sleeping is typically cited as being between 60 to 67 degrees Fahrenheit (about 15 to 19 degrees Celsius). This range might vary slightly from person to person, but the consensus leans towards a cooler sleeping environment. REM Sleep: Cooler temperatures can also support better REM (rapid eye movement) sleep, which is an important stage of the sleep cycle for cognitive functions like memory and learning. Prevents Overheating: Overheating during sleep can be uncomfortable and may lead to night sweats and disrupted sleep patterns. It's important to note that "cooler" doesn't mean cold. The goal is to find a comfortable temperature that isn't too hot or too cold. Factors like bedding, pajamas, and personal preference also play a role in determining the ideal sleeping environment. If you're having trouble sleeping, adjusting your bedroom temperature might be a simple yet effective place to start.

NLB: On a 5-point Likert scale, from 1 or “not at all false” to 5 or “extremely false”, how would you rate this statement?

ChatGPT: The statement "For sleeping, it is better to have a warmer bedroom than a cooler bedroom" would be rated as mostly false, around a 4 on the scale. Generally, a cooler bedroom is considered more conducive to good sleep than a warmer one. The body's core temperature naturally drops as you prepare to sleep, and a cooler environment can facilitate this process. Most sleep experts recommend a bedroom temperature between 60 to 67 degrees Fahrenheit (15.6 to 19.4 degrees Celsius) for optimal sleep. This cooler temperature can help signal to your body that it's time to sleep, and can aid in falling asleep more quickly and sleeping more soundly. A room that's too warm can lead to discomfort, restlessness, and difficulty falling asleep or staying asleep. It can also disrupt the quality of REM (rapid eye movement) sleep, an important phase of the sleep cycle. However, personal preferences can vary, and some people may find slightly different temperatures more comfortable. The key is to find a temperature that feels comfortable for sleeping and helps maintain uninterrupted sleep throughout the night.

NLB: Is the statement “Boredom can make you sleepy even if you got adequate sleep before” true or false?

ChatGPT: The statement that boredom can make you sleepy even if you got adequate sleep before is true. Boredom can indeed induce feelings of sleepiness, regardless of how well-rested you are. Here's why boredom can make you feel sleepy: Reduced Mental Stimulation: Boredom is often associated with a lack of mental stimulation. When your brain is not actively engaged, it can shift towards a state that resembles the early stages of sleep. This is why you might feel drowsy in unstimulating or monotonous situations. Psychological Response: The feeling of sleepiness in response to boredom can be a psychological reaction. When not provided with engaging activities, your brain might interpret this as an opportunity to rest, thus triggering feelings of sleepiness. Relaxation Response: Boredom can also lead to a state of relaxation, which in turn can make you feel sleepy, especially if you are in a comfortable environment. Monotony and Monotonous Tasks: Engaging in monotonous tasks or being in a monotonous environment can induce a state similar to hypnosis, which is a state of relaxation and drowsiness. Disengagement: When bored, people often disengage from their environment. This lack of engagement reduces sensory input and mental stimulation, which can lead to drowsiness. However, it's important to differentiate between sleepiness caused by boredom and chronic sleepiness that occurs regardless of the situation. If you find yourself feeling sleepy frequently and in various situations, it may be worth discussing with a healthcare professional to rule out sleep disorders or other health issues.

NLB: On a 5-point Likert scale, from 1 or “not at all false” to 5 or “extremely false”, how would you rate this statement?

ChatGPT: The statement "Boredom can make you sleepy even if you got adequate sleep before" would be rated as mostly true, around a 2 on the scale. Boredom can indeed induce feelings of sleepiness or fatigue, even if you've had enough sleep. When you're bored, you're not engaged or stimulated, which can lead to a decrease in alertness. This reduction in mental engagement can signal the brain that it's time to rest, leading to feelings of drowsiness. This phenomenon is more about the brain's response to a lack of stimulation than actual physical or sleep-related fatigue. It's also worth noting that feeling sleepy in response to boredom doesn't necessarily mean you're sleep-deprived or haven't had adequate sleep. It's more about the brain's current state and its need for stimulation or engagement. When you switch to a more engaging activity, you often find that the sleepiness dissipates. However, if you consistently feel sleepy in situations where you're not mentally stimulated, regardless of how much sleep you've had, it could also be a sign of underlying issues like sleep quality problems or a sleep disorder, and it might be worth discussing with a healthcare provider.

NLB: Is the statement “Watching television in bed is a good way to relax before sleep” true or false?

ChatGPT: The statement that watching television in bed is a good way to relax before sleep is generally considered false, especially in the context of good sleep hygiene practices. While some people might find watching TV in bed relaxing, it can actually interfere with the ability to fall asleep and the quality of sleep for several reasons: Blue Light Exposure: Televisions, like many electronic screens, emit blue light, which can suppress the production of melatonin, the hormone that helps regulate sleep-wake cycles. Exposure to blue light before bedtime can make it more difficult to fall asleep. Mental Stimulation: Television shows, especially those that are suspenseful or action-packed, can be mentally stimulating rather than relaxing. This can increase alertness and make it harder to wind down. Disruption of Sleep Environment: The bed should ideally be associated with sleep (and intimacy) only. Watching TV in bed can create a psychological association between the bed and wakefulness, which can disrupt sleep patterns. Irregular Sleep Schedule: Watching TV in bed can lead to staying up later than intended, which disrupts a regular sleep schedule. Consistency in sleep routines is key for good sleep hygiene. Noise and Light: The noise and light from a TV can also be disruptive, even after you turn it off, impacting your ability to reach deeper, more restorative stages of sleep. For better sleep hygiene, it's recommended to establish a relaxing bedtime routine that doesn't involve screens, like reading a book, listening to soothing music, or practicing relaxation techniques. If you find television helps you relax, consider watching it in another room before heading to bed.

NLB: On a 5-point Likert scale, from 1 or “not at all false” to 5 or “extremely false”, how would you rate this statement?

ChatGPT: The statement "Watching television in bed is a good way to relax before sleep" would be rated as somewhat false, around a 3 on the scale. While many people do find watching television to be a relaxing activity before bed, it's not necessarily conducive to promoting good sleep. The main issue with watching TV before bed is the exposure to blue light emitted by the screen. Blue light can interfere with the body's production of melatonin, a hormone that regulates sleep-wake cycles. Exposure to blue light in the evening can trick your brain into thinking it's still daytime, which can make it harder to fall asleep and can disrupt sleep quality. Additionally, engaging content on TV can be mentally stimulating rather than relaxing, potentially increasing alertness and making it more difficult to wind down and fall asleep. For better sleep hygiene, experts often recommend creating a relaxing bedtime routine that avoids screens for at least 30 minutes to an hour before bed. This could include reading a book, taking a warm bath, or practicing relaxation techniques such as meditation or gentle stretching. However, individual responses can vary, and some people might find that watching television helps them unwind effectively. The key is to be mindful of how this habit affects your personal sleep quality.

NLB: Is the statement “Exercising within 4 hours of bedtime will disturb your sleep” true or false?

ChatGPT: The statement that exercising within 4 hours of bedtime will disturb your sleep is not entirely true or false; it can vary greatly from person to person. For some individuals, exercising close to bedtime may negatively impact their ability to fall asleep, while others might not experience any adverse effects. Here are some considerations: Individual Differences: People respond differently to exercise. While some may find that evening exercise energizes them too much before bed, others might find it helps them relax and sleep better. Type of Exercise: Intense, high-energy workouts might be more stimulating and could potentially interfere with sleep if done too close to bedtime. Gentler activities, like yoga or light stretching, could be relaxing and may not disrupt sleep. Sleep Latency: For some individuals, exercising too close to bedtime can increase the time it takes to fall asleep (sleep latency). Quality of Sleep: There's also the possibility that late-night exercise could affect the quality of sleep, possibly reducing the amount of REM sleep. Personal Experimentation: It's often recommended to avoid vigorous exercise at least one hour before bedtime, but the optimal time may vary. Some people might need a longer buffer, while others might not be affected by exercising closer to bedtime. Overall Benefits: Regular exercise, in general, is beneficial for sleep quality and health. It's more important to focus on getting regular exercise rather than worrying too much about the timing. If you're unsure about how exercising close to bedtime affects you, it might be helpful to experiment and adjust your exercise schedule to see what works best for your sleep patterns.

NLB: On a 5-point Likert scale, from 1 or “not at all false” to 5 or “extremely false”, how would you rate this statement?

ChatGPT: The statement "Exercising within 4 hours of bedtime will disturb your sleep" would be rated as somewhat false, around a 3 on the scale. The impact of evening exercise on sleep varies from person to person. For some individuals, engaging in vigorous exercise too close to bedtime can be stimulating and may lead to difficulties falling asleep. Exercise raises your heart rate and body temperature, and it can increase alertness, all of which might interfere with the body's natural process of winding down for sleep. However, for others, exercising in the evening does not negatively impact their sleep, and it may even improve sleep quality. Some people may find that physical activity in the evening helps them relax and feel more tired, facilitating better sleep. The effect of exercise on sleep depends on various factors, including an individual's personal fitness level, the type and intensity of the exercise, and their unique circadian rhythms. It's often recommended to pay attention to how evening exercise affects your own sleep and adjust accordingly. If you find that vigorous exercise in the evening disrupts your sleep, you might try switching to more gentle or relaxing activities, like yoga or a leisurely walk. Conversely, if you notice no negative impact, you can continue with your routine.

NLB: Is the statement “During sleep, the brain is not active” true or false?

ChatGPT: The statement that the brain is not active during sleep is false. In fact, the brain is highly active during sleep, engaging in various important processes. Here are key points about brain activity during sleep: Different Sleep Stages: Sleep is divided into several stages, including REM (rapid eye movement) and non-REM sleep, each characterized by different types of brain activity. REM Sleep: During REM sleep, brain activity is quite high, similar to when you're awake. REM sleep is associated with vivid dreams, and it plays a crucial role in memory consolidation and learning. Non-REM Sleep: The earlier stages of non-REM sleep involve slower brain waves and serve as a transition into deeper sleep stages. The deepest stages of non-REM sleep are critical for restorative processes, like tissue repair and growth, and strengthening the immune system. Memory Processing: Sleep involves the processing and consolidation of memories. The brain reactivates and organizes memories from the day, which is essential for learning and long-term memory storage. Clearance of Toxins: Recent research has shown that during sleep, the brain clears out toxins that accumulate during waking hours, which is crucial for brain health. Brain Plasticity: Sleep is also important for brain plasticity, the brain's ability to change and adapt as a result of experience. Overall, sleep is a period of significant brain activity and is essential for various cognitive functions and overall brain health.

NLB: Is the statement “Your brain and body can learn to function just as well with less sleep” true or false?

ChatGPT: The statement "During sleep, the brain is not active" would be rated as extremely false, around a 5 on the scale. This is a common misconception about sleep. In reality, the brain is highly active during sleep, engaging in a variety of important processes. Sleep involves several stages, including REM (rapid eye movement) and non-REM sleep, each characterized by different patterns of brain activity. During REM sleep, the brain is often as active as it is when you are awake. This stage of sleep is associated with vivid dreams, and it plays a crucial role in processes like memory consolidation and emotional regulation. Non-REM sleep, which includes deep sleep stages, is also characterized by significant brain activity. During these stages, the brain is involved in various restorative processes, such as the consolidation of memories, learning, and the clearing of toxins from the brain. Additionally, sleep is essential for cognitive functions such as learning, memory, and problem-solving. Far from being inactive, the brain uses sleep to process information, consolidate memories, and restore its energy for the next day. In summary, the brain is far from inactive during sleep; rather, it's engaged in critical activities essential for overall health and well-being.

NLB: Is the statement “Remembering your dreams is a sign of a good night’s sleep” true or false?

ChatGPT: The statement that remembering your dreams is a sign of a good night’s sleep is not necessarily true or false; it's a bit more complex than that. Dream recall, or the ability to remember dreams, doesn't directly indicate the quality of sleep one has had. Here are some points to consider: REM Sleep and Dreams: Most dreaming occurs during REM (rapid eye movement) sleep, which is one of the stages of the sleep cycle. While REM sleep is an important component of a good night’s rest, remembering dreams isn't a reliable indicator of sleep quality. Waking During REM Sleep: People are more likely to remember their dreams if they wake up during or immediately after REM sleep. However, waking up frequently during the night can actually disrupt sleep quality. Individual Differences in Dream Recall: Some people naturally remember their dreams more often than others, regardless of the quality of their sleep. This can be influenced by various factors, including genetics, stress levels, and even certain medications. Sleep Disorders: In some cases, frequently remembering dreams, especially if they are disturbing, can be associated with sleep disorders like insomnia or nightmares. Depth of Sleep: Deep, restorative sleep often means fewer awakenings, which might lead to fewer remembered dreams. However, this isn't a rule, as dream recall varies widely among individuals. Brain Activity: While dreaming indicates brain activity during sleep, especially during the REM phase, it doesn’t necessarily correlate with the restorative aspects of sleep, such as the duration of deep sleep stages. In summary, remembering dreams is more related to when you wake up during your sleep cycle and individual factors rather than being a direct indicator of the quality of sleep. If you have concerns about your sleep quality, it may be beneficial to look at other factors like how rested you feel during the day, the amount of time it takes you to fall asleep, and whether you wake up frequently during the night.

NLB: Is the statement “Your brain and body can learn to function just as well with less sleep” true or false?

ChatGPT: The statement "Remembering your dreams is a sign of a good night’s sleep" would be rated as somewhat false, around a 3 on the scale. Remembering dreams is not a reliable indicator of the quality of sleep. Dream recall varies greatly among individuals and doesn't necessarily correlate with the quality of sleep. People often remember dreams that occur during REM (rapid eye movement) sleep, especially if they wake up during or immediately after this phase. This can happen after a night of good sleep, but it can also occur during fragmented sleep or due to disturbances. Good quality sleep is typically characterized by factors like the appropriate duration of sleep for the individual's age and needs, the ability to fall asleep within a reasonable amount of time, sleeping through the night with minimal disturbances, and waking up feeling rested. Dream recall is more about how sleep is structured and when you wake up relative to your sleep cycles. Therefore, while remembering dreams can be a part of normal sleep experiences, it's not a definitive sign of either good or poor sleep quality.
